# Determination of the triple oxygen and carbon isotopic composition of CO_2_ from atomic ion fragments formed in the ion source of the 253 Ultra high‐resolution isotope ratio mass spectrometer

**DOI:** 10.1002/rcm.8478

**Published:** 2019-08-05

**Authors:** Getachew A. Adnew, Magdalena E.G. Hofmann, Dipayan Paul, Amzad Laskar, Jakub Surma, Nina Albrecht, Andreas Pack, Johannes Schwieters, Gerbrand Koren, Wouter Peters, Thomas Röckmann

**Affiliations:** ^1^ Institute for Marine and Atmospheric research Utrecht (IMAU) Utrecht University The Netherlands; ^2^ Centre for Isotope Research University of Groningen The Netherlands; ^3^ Geoscience Center Göttingen Georg‐August‐University Göttingen Germany; ^4^ Thermo Fisher Scientific Bremen Germany; ^5^ Department of Meteorology and Air Quality Wageningen University The Netherlands

## Abstract

**Rationale:**

Determination of δ^17^O values directly from CO_2_ with traditional gas source isotope ratio mass spectrometry is not possible due to isobaric interference of ^13^C^16^O^16^O on ^12^C^17^O^16^O. The methods developed so far use either chemical conversion or isotope equilibration to determine the δ^17^O value of CO_2_. In addition, δ^13^C measurements require correction for the interference from ^12^C^17^O^16^O on ^13^C^16^O^16^O since it is not possible to resolve the two isotopologues.

**Methods:**

We present a technique to determine the δ^17^O, δ^18^O and δ^13^C values of CO_2_ from the fragment ions that are formed upon electron ionization in the ion source of the Thermo Scientific 253 Ultra high‐resolution isotope ratio mass spectrometer (hereafter 253 Ultra). The new technique is compared with the CO_2_‐O_2_ exchange method and the ^17^O‐correction algorithm for δ^17^O and δ^13^C values, respectively.

**Results:**

The scale contractions for δ^13^C and δ^18^O values are slightly larger for fragment ion measurements than for molecular ion measurements. The δ^17^O and Δ^17^O values of CO_2_ can be measured on the ^17^O^+^ fragment with an internal error that is a factor 1–2 above the counting statistics limit. The ultimate precision depends on the signal intensity and on the total time that the ^17^O^+^ beam is monitored; a precision of 14 ppm (parts per million) (standard error of the mean) was achieved in 20 hours at the University of Göttingen. The Δ^17^O measurements with the O‐fragment method agree with the CO_2_‐O_2_ exchange method over a range of Δ^17^O values of −0.3 to +0.7‰.

**Conclusions:**

Isotope measurements on atom fragment ions of CO_2_ can be used as an alternative method to determine the carbon and oxygen isotopic composition of CO_2_ without chemical processing or corrections for mass interferences.

## 1 INTRODUCTION

Oxygen has three stable isotopes,^16^O,^17^O and ^18^O, with average terrestrial abundances of 99.76%, 0.04% and 0.21%, respectively. These abundances can be changed by kinetic and equilibrium fractionation processes and other physicochemical effects. Variations in isotopic abundance are reported as deviations of a heavy‐to‐light isotope ratio in a sample relative to a reference material. In the case of oxygen isotopes, the two isotope ratios are ^18^R = [^18^O]/[^16^O] and ^17^R = [^17^O]/[^16^O] and the international standard is Vienna Standard Mean Ocean Water (VSMOW).

(1) $$\delta^{18}O=\frac{{}^{18}R_{\text{sample}}}{{}^{18}R_{\text{VSMOW}}}-1$$

(2) $$\delta^{17}O=\frac{{}^{17}R_{\text{sample}}}{{}^{17}R_{\text{VSMOW}}}-1$$

Since isotope variations are small, they are usually reported in per mill (‰). Most isotope fractionation processes depend on mass. For oxygen isotopes, this results in fractionation patterns where the fractionation in ^17^O is approximately half of the fractionation in ^18^O (Equation 3).

(3) $$\ln\left(\delta^{17}O+1\right)=\lambda \ln\left(\delta^{18}O+1\right)$$

The factor λ $\left(\mathrm{i.e.}\;\frac{{}^{17}R}{{}^{17}R_{\mathrm{ref}}}={\left(\frac{{}^{18}R}{{}^{18}R_{\mathrm{ref}}}\right)}^{\lambda }\right)$ ranges from 0.5 to 0.53 for such mass‐dependent fractionation processes.^1^, ^2^, ^3^ Ozone photochemistry is a well‐known exception to this rule, and O_3_ and related gases have a large oxygen isotope anomaly, expressed as Δ^17^O and referred to as mass‐independent fractionation. We use the logarithmic definition to calculate Δ^17^O of CO_2_ (Equation 4).^2^, ^4^, ^5^ Note that the choice of λ is arbitrary since a variety of sources contribute to the isotopic composition of tropospheric CO_2_ with different fractionations and different three‐isotope slopes. In this study we used a λ value of 0.528 to calculate the Δ^17^O of CO_2_ following Barkan and co‐workers^6^, ^7^ and the ^17^O‐correction algorithm by Brand et al.^8^

(4) $$\Delta^{17}O=\ln\left(\delta^{17}O+1\right)-\lambda \ln\left(\delta^{18}O+1\right)$$

Since the discovery of mass‐independent fractionation,^9^ the Δ^17^O value has been used to study sources/sinks of atmospheric trace gases and chemical reaction pathways. Several studies have shown that CO_2_ acquires Δ^17^O from O_3_ via photochemical isotope exchange in the stratosphere.^10^, ^11^, ^12^, ^13^, ^14^, ^15^, ^16^, ^17^ When this CO_2_ re‐enters the troposphere^18^, ^19^, ^20^ the Δ^17^O is successively reduced by oxygen isotope exchange with leaf, soil and ocean water. Isotopic exchange of CO_2_ with leaf water is more efficient than with ocean water due to the presence of carbonic anhydrase in the leaves, and as a result the main sink for the Δ^17^O of CO_2_ is exchange with leaf water. Precise measurements of the Δ^17^O of CO_2_ may therefore help to better constrain the exchange of CO_2_ between the atmosphere and the biosphere/hydrosphere. For several processes it has been shown that Δ^17^O is a more suitable tracer than the δ^18^O value alone.^21^, ^22^, ^23^, ^24^

Determination of Δ^17^O in CO_2_ with traditional isotope ratio mass spectrometry techniques remains challenging due to the isobaric interference of ^13^C^16^O^16^O (exact mass 44.9932) and ^12^C^17^O^16^O (exact mass 44.9940). Resolving these two isotopologues requires a mass resolving power (m/Δm) of ~56,000, far beyond the resolving power of most traditional mass spectrometer systems. Different alternative techniques have been developed to measure the δ^17^O value of CO_2_: (1) CO_2_ fluorination and isotopic measurement of the released O_2_ 25; (2) conversion of CO_2_ into H_2_O and CH_4_ followed by H_2_O fluorination and isotopic measurement of the released O_2_ 26; (3) isotope exchange between CO_2_ and CeO_2_ 27, 28, 29 or CuO^30^ with known oxygen isotopic composition and measurement of the δ^45^CO_2_ value before and after exchange to calculate the δ^17^O value of CO_2_; (4) isotope exchange between CO_2_ and CeO_2_ followed by isotope analysis of the equilibrated CeO_2_ by laser fluorination^31^; (5) equilibrium exchange of CO_2_ with H_2_O followed by fluorination of H_2_O and measurement of the isotopic composition of released O_2_ 6, 32; (6) isotope exchange between CO_2_ and O_2_ over hot platinum and measurement of the isotopic composition of oxygen before and after exchange to calculate the δ^17^O value of CO_2_.^7^, ^33^ All these methods require either chemical conversion or isotope exchange, which can introduce procedural errors. In recent years, laser‐based absorption spectroscopy techniques to determine δ^17^O values and other isotope signatures of CO_2_ from air samples have been developed.^34^, ^35^, ^36^

Very small variations in the δ^13^C value are used to quantify fluxes between atmosphere and hydrosphere and/or ocean^37^, ^38^, ^39^, ^40^, ^41^. Due to the mass interference of ^12^C^17^O^16^O and ^13^C^16^O^16^O,^8^, ^40^, ^42^, ^43^, ^44^, ^45^, ^46^ the measurements of δ^13^C values require an appropriate correction for ^17^O‐interference. Different “^17^O correction” algorithms are in use to correct for the interference of ^12^C^17^O^16^O on the value of δ^13^C, causing discrepancies between different correction algorithms used. The discrepancies in the δ^13^C value introduced by different ^17^O correction algorithms (i.e. different λ, ^17^R, ^13^R) are explored by Assonov and Brenninkmeijer^42^ in detail. They reported a discrepancy of 0.058‰ for tropospheric CO_2_ with δ^45^(CO_2_) and δ^46^(CO_2_) values of −9.2‰ and +2.180‰ vs NBS19‐CO_2_ between the algorithm by Allison et al^47^ and that by Santrock et al^45^ due to differences in the values of ^17^R and λ. The discrepancies introduced by ^17^O correction algorithms depend on the δ^46^(CO_2_) values^44^ resulting in a different ^17^O correction for CO_2_ having the same δ^45^(CO_2_) value but a different δ^46^(CO_2_) value. By design, most of the ^17^O correction algorithms do not consider the Δ^17^O of the CO_2_ and the ones that do include Δ^17^O require precise measurement of the δ^17^O value of CO_2_. For instance, the algorithm of Allison et al^47^ introduces an error ranging from −0.78 to −0.13‰ for stratospheric CO_2_. Nevertheless, the error introduced to the δ^13^C value because of the use of different values of λ is different for CO_2_ with different Δ^17^O even if the same algorithm is used. It is desirable to use an alternative technique that enables the determination of the δ^13^C value without a bias introduced due to the ^17^O correction algorithm for better use of the δ^13^C values as a tracer to quantify fluxes between atmosphere and hydrosphere and ocean.

Recently developed high‐resolution isotope ratio mass spectrometers^48^, ^49^ are designed to overcome limitations of traditional isotope ratio mass spectrometer systems in terms of mass resolution and sensitivity. In this study, we present a technique to determine the isotope composition of CO_2_ from the C^+^ and O^+^ fragment ions, which are produced from CO_2_ in the ion source of two 253 Ultra (Thermo Fisher Scientific, Bremen, Germany) instruments installed at Utrecht University and the University of Göttingen.

Isotope measurement of fragment ions is not a new concept. The method has been deployed, for example, to study the intramolecular distribution of ^15^N^+^ in N_2_O,^50^, ^51^, ^52^, ^53^, ^54^ to determine the site‐specific carbon isotopic composition of propane^55^ and to measure sulfur isotope ratios in COS.^56^

Here we establish an analytical method to determine the δ^17^O, δ^18^O and δ^13^C values of CO_2_ directly on the C^+^ and O^+^ fragment ions of CO_2_ without any chemical manipulation of the CO_2_ molecule. Notably, this method provides an independent technique to measure Δ^17^O of CO_2_ and the results are validated by comparison with the existing CO_2_‐O_2_ exchange method and by measuring CO_2_ with known Δ^17^O.

## 2 EXPERIMENTAL

### 2.1 The 253 Ultra instrument

The 253 Ultra is the commercial version of a high mass resolution gas source multi‐collector mass spectrometer, which was pioneered with the MAT 253 Ultra prototype in 2012.^48^, ^57^ The high mass resolution of the 253 Ultra enables the investigation of the abundance of isotopologues that suffer from isobaric interferences. The mass resolving power of the instrument can be tuned to m/Δm >35,000 and the peak stability over time is <5 ppm in mass; m/Δm is the width of a peak flank between 5% and 95% of the maximum peak signal. The instrument is controlled by the Qtegra™ software package (Thermo Fisher Scientific).

The ion source of the 253 Ultra is connected to a sample introduction system of four variable volume reservoirs that can be filled with sample or reference gases. The control of the ion source chemistry (adduct formation, fragmentation, formation of metastable ions, linearity and exchange reactions of the sample gas with adsorbed species at the inner ion source surfaces) is critical for accurate isotope ratio measurements. The differentially pumped ion source can be baked to high temperature and is fitted with a variable ion source conductance (VISC) window to adjust the source pumping conductance and to control the residence time of the sample gas in the ionization volume, which is one critical parameter for ion source chemistry. The source slit can be switched to three different slit sizes for low‐, medium‐ and high‐resolution settings. For the instruments at Utrecht University and the University of Göttingen the slit widths are 250 μm, 16 μm and 5 μm. The intermediate aperture at the entrance of the magnetic sector allows an extra‐high‐resolution mode to be selected to achieve m/Δm >35,000 mass resolving power. It should be noted that higher resolution comes at the cost of lower ion beam intensities.

**Figure 1:**
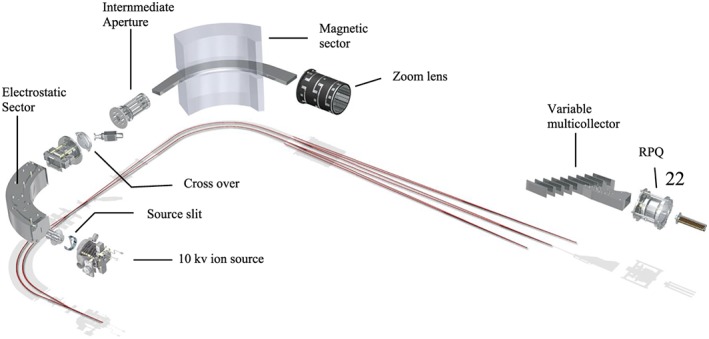
Ion optical layout of the Thermo Scientific 253 Ultra high‐resolution isotope ratio mass spectrometer. In the ion source, the ions are accelerated to 5 keV onto the source slit. After the electrostatic analyzer the ions are accelerated to 10 keV just before passing the crossover. The switchable intermediate aperture behind the magnetic sector is used for extra high mass resolution settings and the zoom lens allows for fine adjustments of peak overlap. The variable multicollector assembly is mounted on the focal detector plane of the mass spectrometer system. The RPQ filter lens discriminates for scattered ions and reduces abundance sensitivity. It is located behind the focal plane right in front of the ion counting detector [Color figure can be viewed at wileyonlinelibrary.com]

The basic setup of the instrument follows a double‐focusing Nier Johnson geometry with a 90^o^ deflection angle of the electrostatic sector (*r* = 22.4 cm) and the magnetic sector (*r* = 23 cm) as shown in Figure 1. Double focusing means that there is stigmatic focusing of the ions passing the source slit regardless of the angular and energy distribution in the ion beam. Usually low‐resolution sector mass analyzers are of the single‐focusing type, i.e. just a magnetic sector. The mass resolving power of a single‐focusing system is limited by the chromatic aberration caused by the energy spread of the ions generated in the ion source. Double focusing can overcome this limitation. In a properly designed double‐focusing system the electrostatic sector optics match the chromatic aberrations of the magnetic sector optics such that the combined system eliminates both, the angular and the chromatic aberrations up to the second order.^58^

In the 253 Ultra the ions are generated at a potential of 10 kV. The ions are accelerated to the source slit of the double‐focusing mass analyzer at a kinetic energy of 5 keV. After passing through the electrostatic analyzer the ions are further accelerated to 10 keV kinetic energy before they pass through the magnetic sector where the ion trajectories are split up according to their mass. Finally, the ions are focused along the focal detector plane of the mass analyzer. The two‐stage acceleration of the ion beam allows a very compact design of the electrostatic sector geometry, which otherwise would have required the radius of the electrostatic sector to be about twice as large as that of the magnetic sector. Due to its compact geometry, the ion optical setup of the 253 Ultra fits onto just one monolithic base plate. The resonance frequency of this rigid mechanical construction is very high and precise, which makes the system robust against low‐frequency vibrations that usually occur in buildings. In order to achieve ultimate stability, the complete mass analyzer and the electronics are housed in a shielded temperature‐stabilized cabinet to be robust against temperature fluctuations in the lab (±2°C).

The variable detector array supports eight moveable detector platforms, which are equipped with Faraday detectors that can be read out with selectable resistors with resistances between 3 × 10^8^ Ω and 10^13^ Ω. The three collector platforms at the high mass end are additionally equipped with compact discrete dynode ion counting detectors^59^ next to the Faraday detectors. The axial detector channel is fixed in position and supports a dual‐detector arrangement, where the ion beam can be switched between a Faraday cup and an ion‐counting channel. The axial ion‐counting detector is equipped with a retardation lens (RPQ‐lens) to reject scattered background ions originating from scattering events along the ion optical flight path (apertures, residual gas particles) which leads to an abundance sensitivity in the ppb range.^48^

### 2.2 Characterization of the 253 Ultra for CO_2_ measurement

We investigated the effect of equilibration time, emission current, source conductance and signal intensity on the ionization of CO_2_ as suggested by Verkouteren et al^58^, ^60^ and Meijer et al.^61^ We characterized the scale contraction effect of the ion source of the 253 Ultra at Utrecht University using two CO_2_ gases (G1 and SCOTT, see Table 1 for details). The characterization of the instrument is performed at low resolution (250 μm entrance slit width, m/Δm ~2000) with five Faraday collectors that are read out with resistors of 3 × 10^8^ Ω, 1 × 10^9^ Ω, 3 × 10^10^ Ω, 1 × 10^11^Ω and 1 × 10^11^ Ω for *m/z* 44, 45, 46, 47 and 48. The corresponding collectors used for this measurement are L2, L1, Center, H1 and H2 for *m/z* 44, 45, 46, 47 and 48, respectively. Here, only data corresponding to *m/z* 44 to 46 are presented. The ion signal of the high intensity ion beam (*m/z* 44) is adjusted before each acquisition to 3.2 × 10^11^ cps (counts per second) with an allowed difference of 1 × 10^10^ cps between the two bellows that are used for the measurement. Under these conditions the ion source pressure is 2.5 × 10^−7^ mbar. The reference measurement is performed with 9.9 kV accelerating voltage, filament emission current of 1.8 mA, equilibration time of 60 s, integration time of 67.1 s and with the VISC window closed.

**Table 1:** Overview of names, suppliers and isotopic compositions of the CO_2_ and O_2_ working standards used in this study. All the CO_2_ gases used have a purity of 99.995% and O_2_ gases have a purity of 99.9998%

CO_2_ working reference gases			
Name	Supplier	δ^13^C vs VPDB [‰]	δ^18^O vs VSMOW [‰]
G1	Air Products, Germany	−39.47 ± 0.012	4.843 ± 0.013
G2	Linde Gas, The Netherlands	−31.733 ± 0.008	34.998 ± 0.023
G5	Air Products, Germany	−10.445 ± 0.010	30.404 ± 0.020
SCOTT	Air Products, Germany	−2.900 ± 0.011	25.803 ± 0.015

O_2_ working reference gases			
Name	Supplier	δ^17^O vs VSMOW	δ^18^O vs VSMOW
IMAU‐O2	Air Products, The Netherlands	9.254 ± 0.007	18.542 ± 0.008
GU‐O_2_	Air Products, Germany	3.849 ± 0.017	8.218 ± 0.007

To study the effect of equilibration time and source conductance, we measure the two gases with equilibration times of 10, 20, 30, 40, 50, 60 and 90 s with the VISC window open and closed. The effect of the emission current is quantified by setting the emission current to 1 mA, 1.5 mA and 1.95 mA. To investigate the effect of signal intensity (cps for *m/z* 44), three experiments with 2.5 × 10^11^ cps, 1.5 × 10^11^ cps and 9 × 10^10^ cps for *m/z* 44 are performed. Note that measurements to characterize the effect of emission control current and signal intensity are performed with an equilibration time of 30 s, so they cannot be directly compared with the reference measurement with an equilibration time of 60 s. The effect of cross contamination is calculated according to Meijer et al^61^ using Equation 5. To calculate the change in scale contraction with changes in equilibration time, we compare the relative difference of the two gases (in δ^13^C and δ^18^O values) measured at different equilibration times with the value obtained at 90‐s equilibration time. Similarly, the scale contraction due to the emission current is calculated with respect to the results obtained at an emission current of 1 mA. The cross contamination (*η*) is calculated as:

(5) $$\eta_{y}=\frac{\left[\delta_{a}^{y}-\delta_{m}^{y}\right]}{\left[2\delta_{a}^{y}+\delta_{a}^{y}*\delta_{m}^{y}\right]}$$

where *y* is 13 (for δ^13^C) or 18 (for δ^18^O), the index *a* indicates the respective δ value under reference conditions (90‐s equilibration time and 1 mA emission current), and index *m* indicates the δ value at a different equilibration time or different emission current.

To link our results to international isotope scales, we use a set of isotopically different pure O_2_ and CO_2_ reference gases. Multiple aliquots of each gas were sent to Eugeni Barkan from the Hebrew University of Jerusalem (Jerusalem, Israel) for analysis. This research group also provides high‐precision δ^17^O values and has established a direct link between the oxygen isotope scales of O_2_ and CO_2_. The reported results were assigned to our reference gas cylinders, which were also measured extensively on the Thermo Scientific Delta ^Plus^ XL™ instrument in our laboratory and on the 253 Ultra. The appropriate scale contraction factors (see Section 4) are used to convert the raw data into the scale of the Hebrew University of Jerusalem.^6^, ^62^, ^63^

### 2.3 Fragment method

**Figure 2:**
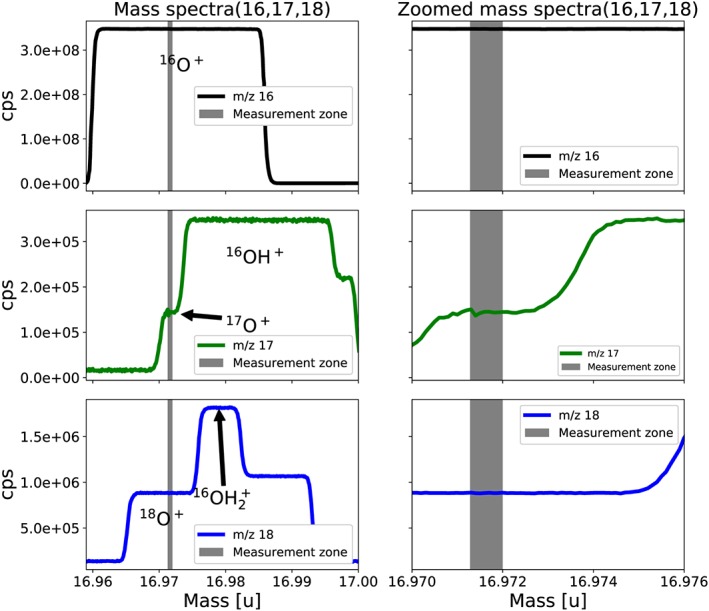
Medium‐resolution mass spectra for measurement of ^16^O^+^, ^17^O^+^ and ^18^O^+^ fragment ions of CO_2_. The shaded area shows the region of the shoulder where ^17^O^+^ is measured interference‐free, a magnified view is shown in the right panels. The mass scale (x‐axis) applies to the middle panels (^17^O) for the top and bottom panels; the mass scale is shifted one mass down or up, respectively [Color figure can be viewed at wileyonlinelibrary.com]

The ^17^O^+^ fragment ion measurements at Utrecht University are performed at medium resolution (16 μm entrance slit width, m/Δm >7500) with the “reference” source settings mentioned above, i.e., emission current of 1.80 mA, accelerating voltage 9.9 kV, VISC window closed. The ion signals are registered in three Faraday collectors (L3, Center, H3) that are read out with resistors of 1 × 10^11^ Ω, 1 × 10^13^ Ω and 1 × 10^13^ Ω for *m/z* 16, 17 and 18, respectively. The ion signal intensity is adjusted before each acquisition to 9.2 × 10^8^ cps on *m/z* 16, which corresponds to a source pressure of ~2.5 × 10^−7^ mbar, with a tolerance of 3 × 10^6^ between the bellows. Reasonable source pressures for fragment ion measurement are determined to fall between 2.0 and 4.5 × 10^−7^ mbar (resulting in major ion beam signals of 0.75 to 1.25 × 10^9^ cps at medium resolution), corresponding to the linear portion of the source pressure vs signal intensity relationship for *m/z* 16 (Figure S1, supporting information). The integration and equilibration times are 67.1 and 60 s, respectively, which implies that in a measurement cycle both sample and reference are measured for 67.1 s out of 254.2 s, i.e., 26% of the time. Figure 2 shows the mass spectra covering the range of *m/z* 16, 17 and 18. The main interference for the ^17^O^+^ ion (mass 16.9991 u) is OH^+^ (mass 17.0027 u). The mass difference between these two ions is only 0.0036 u. With the 253 Ultra, they are sufficiently separated using the medium‐resolution slit to enable measurement of ^17^O^+^ on a narrow plateau without interference from OH^+^. In this study the medium‐resolution slit is chosen since the plateau is sufficiently flat and gives a sufficient signal to allow stable positioning for ^17^O^+^ measurement, as shown in Figure 2. The width of the plateau can in principle be increased by going to high mass resolution, but this would result in a reduction of the ion current by a factor of 3 and a corresponding increase in the required measurement time to reach a certain precision. For ^18^O^+^ (mass 18.9984 u) the mass difference to its main interference H_2_O^+^ (19.0148 u) is 0.0164 u which results in a broad shoulder even at medium mass resolution. The potential effect of other interferences is discussed below.

Small shifts in the mass scale regularly lead to a deterioration of measurement precision, when the mass position shifts away from the small ^17^O^+^ shoulder. This can be largely circumvented by resetting the mass scale at regular time intervals during the measurement. The present version of the Qtegra software does not allow automatic positioning on a shoulder of multiple overlapping peaks. Therefore, the collector configuration is carefully arranged such that the center of the *m/z* 16 peak is precisely located at the shoulder of the *m/z* 18 and *m/z* 17 peaks where ^17^O^+^ and ^18^O^+^ can be measured interference‐free. A peak centering is then performed on *m/z* 16 before each acquisition which is precise enough to relocate the system on the narrow shoulder of the *m/z* 17 peak. Nevertheless, instabilities in the mass scale are still considered a main contributor to the remaining error above counting statistics, and an automatic positioning routine that scans the ^17^O^+^ shoulder directly to reposition the peak might improve the precision.

All ^17^O^+^ fragment ion measurements on the 253 Ultra at the University of Göttingen are performed at medium resolution (16 μm entrance slit width, m/∆m ~7500) with 9.85 kV accelerating voltage and 1.85 mA emission current, with the VISC window closed. The integration and equilibration times are 67.1 and 12 s, respectively, which implies that in a measurement cycle both sample and reference are measured for 67.1 out of 158.2 s, i.e., 42.4% of the time. Three Faraday collectors (L3, Center, H3), equipped with 1 × 10^10^ Ω, 1 × 10^13^ Ω and 1 × 10^12^ Ω resistors, are used to detect the ion signals for *m/z* 16, 17 and 18, respectively. The signal intensity is adjusted per acquisition on *m/z* 16, with a target intensity of 1.2 × 10^9^ cps (tolerance 0.2%), corresponding to a source pressure of 4.12 × 10^−7^ mbar.

**Figure 3:**
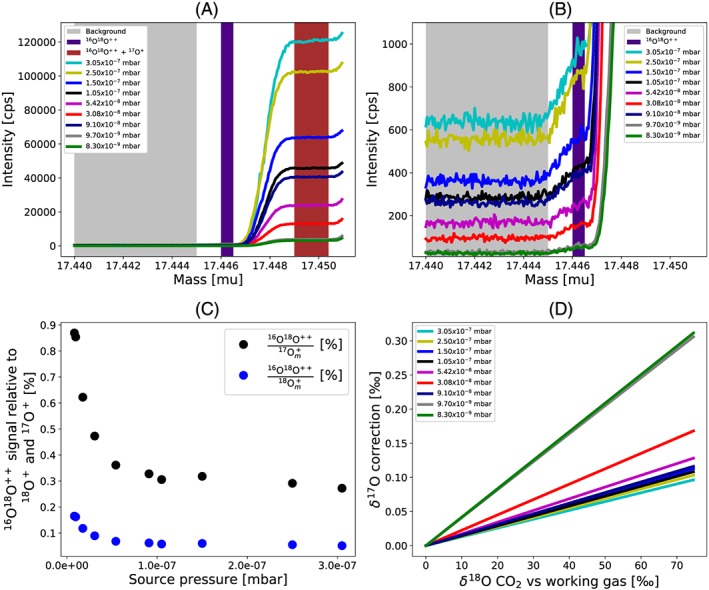
Interference of ^16^O^18^O^++^ on the measurement of the ^17^O^+^ fragment ion. A, Mass spectra at different source pressure. B, Zoom to the background signal where the interference of ^16^O^18^O^++^ can be detected starting around mass 17.445, 0.002 mass units before the larger ^17^O^+^ peak. The CDD background signals determined in the grey shaded area were subtracted from the signals in the dark shaded area to quantify the contribution from ^16^O^18^O^++^. C, Abundance of the ^16^O^18^O^++^ signal relative to the measured signals ^17^O^+^_m_ and ^18^O^+^_m_ (in %). For source pressures above 10^−7^ mbar, where our measurements were carried out, the ^16^O^18^O^++^ signal is 0.06% of the ^18^O^+^ signal, which results in a contribution of 0.3% to the ^17^O^+^ ion beam. D, Bias in the δ^17^O value introduced by ^16^O^18^O^++^ as a function of the difference in the δ^18^O value between sample and working gas for different source pressures [Color figure can be viewed at wileyonlinelibrary.com]

The doubly charged ^16^O^18^O^++^ ion is very close in mass to ^17^O^+^ (Table S5, supporting information) and interferes at the lower mass shoulder of the ^17^O^+^ peak. Figure 3 shows mass spectra recorded at medium resolution using the compact discrete dynode (CDD) collector of the H2 collector unit of the 253 Ultra (H2‐CDD). The interference of ^16^O^18^O^++^ can be detected 0.002 mass units before the larger ^17^O^+^ peak starts. The ^16^O^18^O^++^ ion is formed in the ion source, probably from the recombination of ^16^O and ^18^O atom fragments. Therefore, the contribution of ^16^O^18^O^++^ to ^17^O^+^ depends on the ^18^O content of the gas, and it has to be corrected to avoid a systematic bias in the δ^17^O determination when the δ^18^O values of the sample and the working reference gas are different. Figure 3C shows that the ^16^O^18^O^++^ signal increases relative to the ^17^O^+^ and ^18^O^+^ signals towards lower source pressures but it is quite stable at pressures above 10^−7^ mbar. At 2.5 ×10^−7^ mbar, where our measurements were carried out, the ^16^O^18^O^++^ signal is 0.055% of the ^18^O^+^ signal, which results in a ^16^O^18^O^++^ contribution of about 0.3% to the ^17^O^+^ ion beam. Based on this correction factor, Figure 3D shows the calculated effect of ^16^O^18^O^++^ on the measured δ^17^O values, as a function of the δ^18^O difference between sample and working reference gas and for different source pressures. The correction is probably instrument and tuning‐dependent and should be determined regularly. We applied a corresponding correction to the data where we compare the results from the O‐fragment method and CO_2_‐O_2_ exchange method.

**Figure 4:**
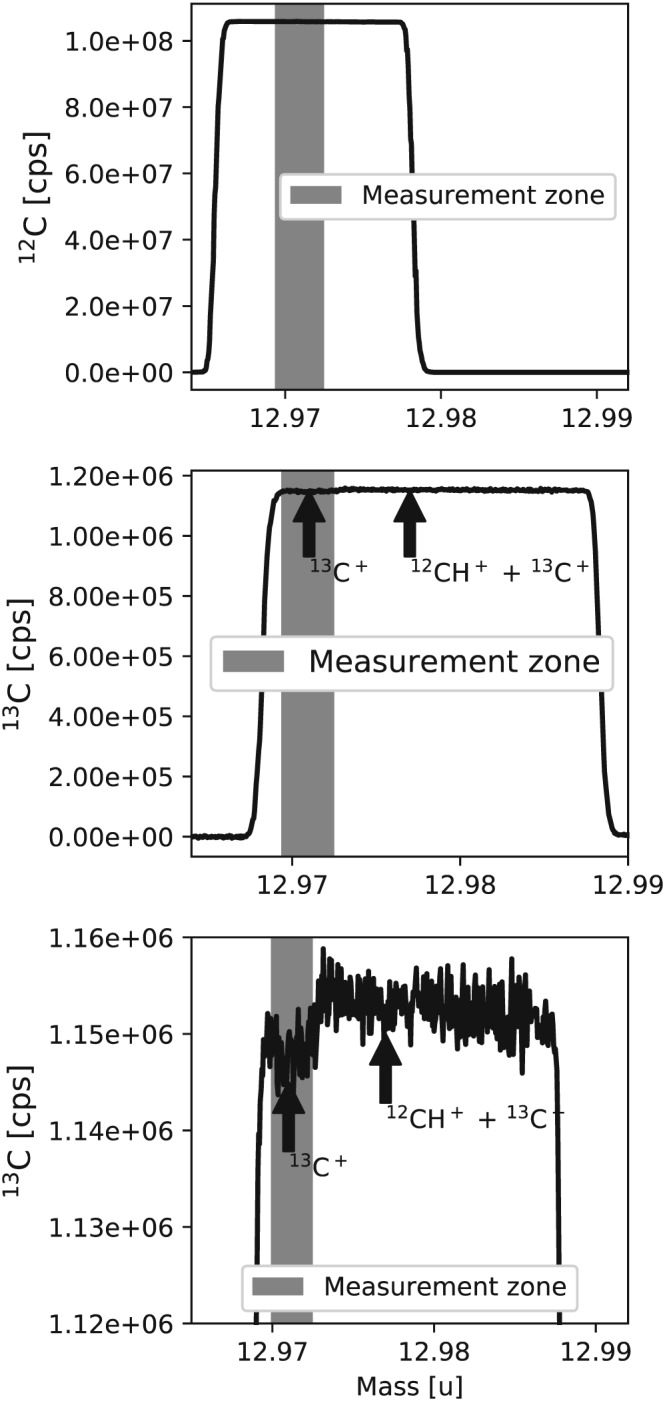
Medium‐resolution mass spectra for measurement of ^12^C^+^ and ^13^C^+^ fragment ions of CO_2_. The shaded area shows the region where the isotope measurements were performed. Measurement of the C fragment is performed at medium resolution. The mass scale (x‐axis) applies to the middle and bottom panels (^13^C); for the top panel, the mass scale is shifted one mass down

The ^13^C^+^ fragment ion is measured at Utrecht University at medium resolution (16 μm entrance slit width) with the same emission current, acceleration voltage, integration time and equilibration time as used for the ^17^O^+^ fragment method, again with the VISC window closed. The ion signals are registered in two Faraday collectors (L4 and Center) that are read out with resistors of 1.0 × 10^11^ Ω and 1.0 × 10^13^ Ω for ^12^C^+^ and ^13^C^+^, respectively. The mass spectra covering the range for ^12^C^+^ and ^13^C^+^ are shown in Figure 4. The main interference for ^13^C^+^ (mass 13.0034 u) is ^12^CH^+^ (mass 13.0078 u), which requires a mass resolving power of 2900. This is well resolved with the medium‐resolution slit of the 253 Ultra (m/Δm >7500).

To establish the scale contraction correction for fragment ion measurements, isotopically well‐characterized pure CO_2_ gases (see section 3.2) were analyzed both with the molecular ion method and with the fragment ion method. The CO_2_ and O_2_ working reference gases used in this study are summarized in Table 1. The two CO_2_ samples, G3 and G4, are prepared from G2 by adding isotopically anomalous CO_2_ generated by UV‐induced isotope exchange between CO_2_ and O_3_.

The reported internal precision of the fragment technique is compared with the expected error (precision) based on counting statistics (EECS), which is calculated as:

(6) $$\text{EECS}=\sqrt{\frac{2}{N*t_{\mathrm{int}}*n}}$$

where *N* is the average count rate (cps), *t*_int_ is the integration time in seconds, *n* is the number of measurement cycles and the factor $\sqrt{2}$ accounts for the fact that the reference and the sample both introduce the same error to the δ value. Throughout the manuscript the error of a single measurement series is reported as the standard error of the mean. When we quantify errors for more than one measurement (series), we report the standard error times the Student's t‐factor to cover the 95% confidence interval.

### 2.4 O_2_‐CO_2_ exchange method

A schematic diagram of the O_2_‐CO_2_ exchange experimental setup at Utrecht University is shown in Figure S2 (supporting information). The central part of the CO_2_‐O_2_ exchange system is the exchange reactor, which is made of quartz, while the other parts are made from borosilicate glass. The general design is similar to the one in Barkan et al,^7^ except for some modifications in the ways of introducing CO_2_ and O_2_ into the reactor.

Approximately 1.7 mL of pure CO_2_ with known (measured) δ^18^O value was expanded to the glass line and trapped cryogenically using liquid nitrogen (LN_2_) in the calibrated volume (CV, 2.319 mL). The amount of CO_2_ was precisely determined with a pressure sensor (PS9504, Geological and Nuclear Sciences Ltd, Lower Hutt, New Zealand). The CO_2_ sample was then transferred cryogenically to the quartz reactor. The trapping in the quartz reactor occurs at the horizontal tube that is continuously cooled using LN_2_ provided by a microdosing system (Norhof 900 series LN_2_ cooling system, Ede, The Netherlands). After introduction of the CO_2_ sample, an approximately equal amount of pure O_2_ (IMAU‐O2) with known δ^17^O and δ^18^O values is admitted to the small volume above the reactor and then expanded into the reactor. The CO_2_ is then released from the cold tube by stopping the LN_2_ microdosing system, and the gases are allowed to react for 30 min in the quartz reactor that contains 0.18 g of platinum sponge (99.9% purity, Sigma Aldrich, St Louis, MO, USA) at the bottom, which is heated to 750°C with a temperature‐controlled oven (CFH VC401A06A‐0000R, Kurval, Nieuw‐Vennep, The Netherlands). After 30 min, CO_2_ is extracted cryogenically in a double U trap, while O_2_ is collected behind this trap on 3 pellets of molecular sieve 13X (1.6 mm, Sigma Aldrich) at LN_2_ temperature. The isotopic composition of the exchanged O_2_ is measured using a dual‐inlet system on the Delta^Plus^XL isotope ratio mass spectrometer (Thermo Fisher Scientific) using three Faraday collectors equipped with resistors of 3 × 10^8^ Ω, 3 × 10^10^ Ω and 3 × 10^11^ Ω for *m/z* 32, 33 and 34, respectively. The value of δ^17^O (CO_2_) is then calculated from the change in the δ^17^O(O_2_) value before (index *i* = “initial”) and after (index *f* = “final”) isotope exchange with CO_2_ based on the following mass balance equation (Eequation 7), after Barkan et al^7^:

(7) $$\delta^{17}O_{i}\left({\mathrm{CO}}_{2}\right)=\frac{1}{\beta }\left[\left(\delta^{17}O_{f}\left(O_{2}\right)+1\right)\left(\alpha^{17}\beta +1\right)-\left(\delta^{17}O_{i}\left(O_{2}\right)+1\right)\right]-1\;$$

where β is the molar ratio of CO_2_ to O_2_ and $\alpha^{17}\left({\mathrm{CO}}_{2}/O_{2}\right)=\frac{\delta^{17}O_{f}\left({\mathrm{CO}}_{2}\right)+1}{\delta^{17}O_{f}\left(O_{2}\right)+1}\;$ and $\alpha^{18}\left({\mathrm{CO}}_{2}/O_{2}\right)=\frac{\delta^{18}O_{f}\left({\mathrm{CO}}_{2}\right)+1}{\delta^{18}O_{f}\left(O_{2}\right)+1}\;$ are the ^17^O and ^18^O equilibrium fractionation factors between CO_2_ and O_2_ in the presence of the hot platinum catalyst.^7^ In our CO_2_‐O_2_ exchange setup the equilibrium fractionation factors are α^17^(CO_2_/O_2_) = 1.0006657 and α^18^(CO_2_/O_2_) = 1.000998, determined by measuring the isotopic composition of CO_2_ and O_2_ after isotope exchange was fully established.

### 2.5 Samples

#### 2.5.1 Preparation of CO_2_ with known δ^17^O and δ^18^O values

At Utrecht University, CO_2_ with known isotopic composition is prepared by combusting a pure graphite rod (99.9995% purity, Alfa Aesar, Part No: 40765) (Thermo Fisher Scientific) in isotopically known pure IMAU‐O2 (Table 1). The graphite rod (3.05 mm × 32 mm) is wrapped in a sheet of platinum foil and platinum wire and placed inside a quartz reactor as shown in Figure S3 (supporting information). The experimental setup is similar to the one presented in Barkan and Luz,^64^ except for a modification in the way that CO_2_ is trapped. The graphite rod is conditioned by heating to 1000°C in vacuum for 2 days. The combustion experiment is performed at 750°C and the CO_2_ is trapped immediately at LN_2_ temperature using a collar trap (Figure S3, supporting information) to avoid fractionation due to possible exchange with the graphite. After the O_2_ has been fully combusted to CO_2_ (as indicated by the pressure), the reactor is cooled to below 200°C and the collar trap is heated to room temperature (25°C) to release the CO_2_. The CO_2_ is collected in a break seal tube at LN_2_ temperature. After each conversion experiment the graphite rod is re‐conditioned by heating at 900°C for 1 h to avoid contamination from remaining oxygen.

At the University of Göttingen, isotopically light CO_2_ was produced from combustion with isotopically depleted O_2_ using a slightly different setup. Instead of using platinum foil and wire as catalyst, the graphite rod was immersed in chloroplatinic acid and dried before being installed in the quartz reactor. Isotopically light oxygen for the reaction was provided by hydrolysis of Antarctic precipitation (Dronning Maud Land, δ^2^H = −341.1‰ vs SMOW and δ^18^O = −42.4‰ vs SMOW). After full combustion, the produced CO_2_ was transferred into a glass vial, which was kept at LN_2_ temperature.

#### 2.5.2 Preparation of ^17^O‐enriched CO_2_

^17^O‐enriched CO_2_ is prepared by inducing oxygen isotope exchange between CO_2_ (G2) and O_2_ (IMAU‐O2) (via O_3_ and O(^1^D))^65^ using a Hg ultraviolet (UV) lamp (Oriel Instruments, Newport Corporation, Stratford, CT, USA). The borosilicate photolysis reactor is equipped with a UV‐transparent Suprasil™ finger in the center to place the lamp, as shown in Figure S4 (supporting information). 50 mbar of CO_2_ is expanded into the 2‐L reactor and O_2_ is then expanded into the reactor until the pressure reading reaches around 1 bar. The mixture is then allowed to photolyze for 18 h without regulating the temperature. Due to the heat produced by the UV light the temperature outside the reactor reaches 30°C during photolysis, and is much higher at the Suprasil finger, but this is only a preparative experiment where the exact conditions are not critical. After photolysis‐induced isotope exchange, CO_2_ is separated cryogenically in a glass spiral trap at LN_2_ temperature and O_2_ is pumped out. Finally, the CO_2_ is collected in a sample vial containing nickel foil (thickness 0.05 mm, 99.98% purity, Goodfellow Cambridge Ltd, Huntingdon, UK). O_3_ that is formed during photolysis is also condensed with CO_2_ and is decomposed to O_2_ by heating the sample vial with a heat gun at 500°C for 10 min. Ni foil catalyzes the decomposition of O_3_ to O_2_. The CO_2_ is then trapped again with LN_2_ and the O_2_ that has formed from O_3_ decomposition is pumped out. Finally, the CO_2_ is passed through a glass U‐trap at dry‐ice temperature (−78°C) to remove remaining traces of water. Heating the O_3_ and CO_2_ mixture above 200°C might cause isotope exchange between O_3_ and CO_2_,^66^ but it does not cause a problem for our purpose which is to prepare ^17^O‐enriched CO_2_.

The isotopic composition of the ^17^O‐enriched CO_2_ sample is measured with the 253 Ultra for both molecular ions (*m/z* of 44 to 46) to determine δ^18^O and δ^13^C values, and atom fragments to measure δ^17^O and δ^18^O values. By diluting the ^17^O‐enriched CO_2_ with pure non‐anomalous CO_2_ from the reference CO_2_ tank (G2), two gas mixtures are prepared with target Δ^17^O values of approximately 0.25‰ and 0.55‰. The two mixtures are finally measured both with the CO_2_‐O_2_ exchange method and with the fragment technique.

## 3 RESULTS

### 3.1 Instrument characterization and scale contraction

Scale contraction decreases with equilibration time and source pressure (signal intensity), when the variable conductance window is fully opened and when the emission current is decreased. A detailed investigation of these parameters is presented in the supporting information (Figures S5, S6, and S7, and Tables S1 and S2, supporting information). The effects of ion source pressure and emission control current are the major contributors to the scale contraction. Scale contraction can be minimized if the measurement is performed at high source pressure, low emission control current and with the VISC window open. The drawback of having a higher source pressure is potentially a reduction in the life time of the filament, while having lower emission control current reduces the ionization of the molecules which leads to a lower signal. We suggest following the recommendations of Verkouteren et al,^60^ to minimize cross contamination in dual‐inlet isotope ratio mass spectrometry measurements. In general, the different parameters affect the δ^18^O and δ^13^C values in the same way, but the effects are larger for the δ^18^O values than for the δ^13^C values. The origin of the qualitatively different behavior for δ^18^O and δ^13^C values could not be identified and requires further study.

By comparing the results of the molecular ion measurements on the 253 Ultra with the values assigned to our reference gases by the Hebrew University of Jerusalem, a scale contraction factor of 0.981 was established and applied for molecular ion measurements. The scale contraction factor is the ratio of the difference between the two CO_2_ gases (G1 and SCOTT) measured with the 253 Ultra at Utrecht University and the assigned relative difference by the Hebrew University of Jerusalem. Thus, the final values reported below are linked to the isotope scale of the Hebrew University of Jerusalem.^6^, ^62^, ^63^

The key parameter relevant for the validation of the fragment ion method is the scale contraction of a fragment ion measurement relative to a molecular ion measurement. This was determined by analyzing a set of three isotopically distinct pure CO_2_ gases both with the traditional CO_2_^+^ method and with the fragment method (both O^+^ and C^+^ fragments). For the traditional molecular ion measurements, the ^17^O‐correction procedure from Brand et al^8^ is used. Table 2 shows that the scale contraction for fragment ion measurements is slightly larger than the one for molecular ion measurements. The scale contraction seems to be also slightly larger for measurements on the C^+^ fragment than for those on the O^+^ fragment, but more measurements are required to quantify this more thoroughly. Note that each individual measurement series presented in Tables 3 and 4 (CO_2_^+^ molecule plus O^+^ fragment and C^+^ fragment) takes one full day. For the evaluation of the Δ^17^O measurements below we use the relative scale contraction of 0.997 determined for the value of δ^18^O between the traditional CO_2_^+^ method and the O‐fragment method (Table 2).

**Table 2:** δ^13^C and δ^18^O scale contraction factors for measurements with the fragment method relative to the traditional measurement technique on molecular ions, using the ^17^O correction algorithm from Brand et al.^8^ Both measurements were carried out on the 253 Ultra using three CO_2_ gases (G1, SCOTT and G2)

Measurement	Fragment (253 Ultra) vs molecule (253 Ultra)	
	δ^13^C	δ^18^O
G1 vs G2	0.996	0.997
G1 vs SCOTT	0.993	0.997
SCOTT vs G2	0.996	0.997
Average ± SE*t	0.995 ± 0.0016	0.997

**Table 3:** Oxygen isotope composition of various CO_2_ reference gases measured with the ^17^O^+^ fragment method. δ^17^O and δ^18^O values are given relative to VSMOW; Δ^17^O is calculated according to Equation 4 using λ = 0.528. Individual errors are standard errors of the mean of the corresponding measurement series. The error for the mean is the standard error of the mean for the six experiments multiplied by Student's t‐factor for the 95% two‐sided confidence. Γ is the ratio between the measured precision and the precision expected from counting statistics for δ^17^O and n is the number of sample‐standard cycles. For δ^18^O, Γ ≈ 1 for individual measurement series, but the weighted mean error is similar to the one for δ^17^O, which indicates additional handling errors in sample introduction at the 0.01‰ level. The values in the parentheses are the isotopic compositions of oxygen used for combustion

Experiment	n	Γ	δ^17^O [‰]	δ^18^O [‰]	Δ^17^O [‰]
Reference CO_2_ [Figure 5A]					
1	227	1.54	15.661 ± 0.037	30.406 ± 0.011	−0.276 ± 0.036
2	109	1.53	15.719 ± 0.048	30.419 ± 0.14	−0.225 ± 0.048
3	47	1.73	15.672 ± 0.082	30.444 ± 0.025	−0.284 ± 0.081
4	109	1.48	15.701 ± 0.047	30.397 ± 0.014	−0.231 ± 0.047
5	169	1.42	15.672 ± 0.038	30.380 ± 0.011	−0.251 ± 0.038
6	68	1.47	15.668 ± 0.057	30.379 ± 0.016	−0.255 ± 0.057
Mean ± SE*t			15.682 ± 0.019	30.404 ± 0.021	−0.254 ± 0.019
Reference O_2_ to CO_2_ [Figure 5B] (vs reference CO_2_)					
1	64	1.1	−10.518 ± 0.028	−19.266 ± 0.017	−0.303 ± 0.026
2	64	0.8	−10.586 ± 0.021	−19.367 ± 0.009	−0.316 ± 0.020
3	64	1.2	−10.639 ± 0.035	−19.360 ± 0.010	−0.373 ± 0.036
4	64	1.1	−10.534 ± 0.027	−19.184 ± 0.009	−0.362 ± 0.028
5	64	1.0	−10.516 ± 0.026	−19.194 ± 0.011	−0.339 ± 0.026
6	64	1.2	−10.743 ± 0.030	−19.595 ± 0.010	−0.352 ± 0.030
7	64	1.2	−10.741 ± 0.030	−19.610 ± 0.007	−0.342 ± 0.030
8	64	1.3	−10.611 ± 0.34	−19.345 ± 0.009	−0.353 ± 0.034
			−10.611 ± 0.062	−19.365 ± 0.109	−0.342 ± 0.016
Reference O_2_ to CO_2_ [Figure 8A]					
1	200	2.43	9.206 ± 0.071	18.510 ± 0.018	−0.520 ± 0.071
2	300	1.99	9.220 ± 0.048	18.539 ± 0.018	−0.522 ± 0.048
3	180	1.88	9.298 ± 0.042	18.495 ± 0.017	−0.423 ± 0.042
4	200	2.16	9.302 ± 0.048	18.465 ± 0.017	−0.403 ± 0.048
Mean ± SE*t			9.256 ± 0.059 (9.254 ± 0.007)	18.503 ± 0.035 (18.542 ± 0.008)	−0.467 ± 0.074 (−0.489 ± 0.008)
Light O_2_ to CO_2_ [Figure 8B]					
1	216	2.13	−26.934 ± 0.097	−50.791 ± 0.024	0.219 ± 0.067
2	208	1.43	−26.611 ± 0.355	−50.075 ± 0.512	0.182 ± 0.059
3	256	1.34	−26.381 ± 0.231	−49.824 ± 0.318	0.311 ± 0.056
Mean ± SE*t			−26.666 ± 0.488 (−26.239 ± 0.002)	−50.329 ± 0.817 (−49.614 ± 0.002	0.237 ± 0.097 (0.279 ± 0.011)

When the appropriate scale correction parameters are applied, the δ^13^C and δ^18^O values obtained from the fragment and molecular ion measurements generally agree at the ~0.01–0.03‰ reproducibility level (except for one outlier in δ^13^C, G1 vs SCOTT = −36.665 ± 0.002‰ and −36.601 ± 0.020‰ for molecular and fragment ion measurements respectively (Figure S10, supporting information). Isotope ratio measurements on C and O fragment ions could be an independent method to validate/evaluate traditional isotope measurements and ion (^17^O) correction algorithms at a level of precision similar to the reported differences between different ion correction schemes.

Figures S8, S9 and S10 (supporting information) show that the fragment method returns the same value when two pure CO_2_ gases are measured directly, and via a third intermediate gas for δ^13^C, δ^18^O and δ^17^O values. Tables 3 and 4 show that isotope ratios based on the ^13^C^+^ and ^18^O^+^ fragment ions are both measured with a precision close to the counting statistics limit.

**Table 4:** Comparison of δ^13^C and δ^18^O values obtained using the C‐fragment and O‐fragment techniques with results from the traditional molecular measurements for pure CO_2_ gases. For the measurements on the molecule, the ^17^O correction according to Brand et al^8^ is used. Γ is the ratio between measured precision and the precision estimated from the counting statistics and n is number of cycles for the fragment measurement

δ^13^C					
Sample	Exp	*n*	Γ	δ^13^C [‰] (^13^C^+^ measurement)	δ^13^C [‰] ^13^CO_2_^+^ measurement
G1vs G2	1	45	1.0	−7.968 ± 0.015	−7.963 ± 0.001
	2	20	0.73	−7.967 ± 0.022	−7.984 ± 0.001
	3	38	0.74	−7.991 ± 0.016	−7.967 ± 0.001
	4				−7.981 ± 0.001
	5				−7.972 ± 0.001
	6				−7.978 ± 0.002
Average ± SE*t				−7.975 ± 0.023	−7.974 ± 0.007
G2 vs SCOTT	1	49	0.84	−28.933 ± 0.015	−28.881 ± 0.001
	2				−28.923 ± 0.001
	3				−28.916 ± 0.001
	4				−28.913 ± 0.001
	5				−28.915 ± 0.001
Average ± SE*t					−28.910 ± 0.016

δ^18^O					
Sample	Exp	n	Γ	δ^18^O (‰) (^18^O^+^ measurement)	δ^18^O (‰) CO_2_^+^ measurement
G1 vs G2	1	145	0.9	−29.106 ± 0.010	−29.140 ± 0.001
	2	146	0.9	−29.138 ± 0.010	−29.146 ± 0.015
	3	107	0.7	−29.125 ± 0.010	−29.132 ± 0.001
	4	81	0.8	−29.128 ± 0.012	−29.101 ± 0.001
	5	143	0.9	−29.086 ± 0.010	−29.093 ± 0.001
	6	89	1	−29.102 ± 0.013	−29.135 ± 0.002
	Average ± SE*t			−29.114 ± 0.016	−29.124 ± 0.018
SCOTT vs G2	1	196	0.7	−8.885 ± 0.010	−8.841 ± 0.001
	2	163	0.9	−8.873 ± 0.010	−8.847 ± 0.001
	3	143	0.8	−8.866 ± 0.010	−8.886 ± 0.002
	4	177	0.9	−8.881 ± 0.010	−8.876 ± 0.002
		139	0.7	−8.835 ± 0.010	−8.876 ± 0.002
	Average ± SE*t			−8.868 ± 0.019	−8.865 ± 0.019

### 3.2 Fragment measurement

- δ^17^O, δ^18^O and Δ^17^O: reproducibility

**Figure 5:**
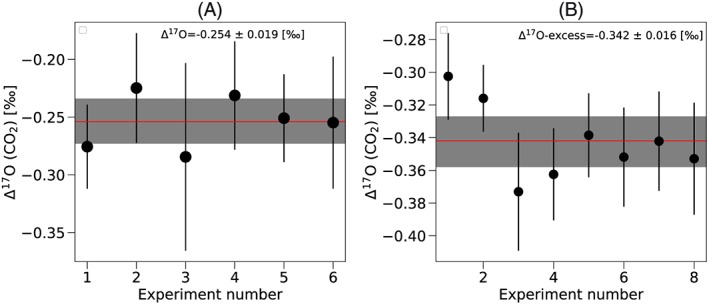
A, Δ^17^O (CO_2_) measured with the O‐fragment method for a pure CO_2_ (G5, see Table 1), measured at Utrecht University. B, Δ^17^O (CO_2_) measured with the O‐fragment method for CO_2_ prepared by combusting graphite rod with pure O_2_ (GU‐O_2_) (δ^17^O = −10.611 ± 0.062‰ and δ^18^O = −19.365 ± 0.109‰, relative to the working standard) measured at the University of Göttingen. Error bars represent ±1 standard error of the mean (SEM). The red line shows the mean and the shaded area is the SEM times Student's t‐factor (95% confidence) [Color figure can be viewed at wileyonlinelibrary.com]

Figure 5A shows Δ^17^O for a pure CO_2_ (G5) sample with six replicates measured using the O‐fragment method at Utrecht University. The δ^17^O and δ^18^O values of the CO_2_ are given in Table 3. The measurement times are between 3 and 12 h. The δ^17^O values are measured with an individual measurement error (standard error of the mean) ranging from 37 to 82 ppm, while the δ^18^O values have an individual measurement error of 11 to 25 ppm (standard error of the mean, SEM). The measurement precision for the δ^17^O values is worse than that expected from counting statistics by a factor of 1.42 to 1.73. As shown in Figure 5A and Table 3, from these six replicates the Δ^17^O reproducibility is 19 ppm (standard error times Student's t‐factor for 95% confidence). At the University of Göttingen the reproducibility experiment is performed using CO_2_ produced by combustion of a graphite rod with pure O_2_ (GU‐O_2_) (Figure 5B). The δ^17^O and δ^18^O values of the CO_2_ are given in Table 3 relative to the working reference. The δ^17^O values are measured with an individual measurement error (SEM) ranging from 21 to 35 ppm while the δ^18^O values have an individual measurement error of 7 to 17 ppm (SEM). As shown in Figure 5B and Table 3, from these eight replicates the Δ^17^O reproducibility is 16 ppm (standard error times Student's t factor for 95% confidence). The reproducibility for the δ^17^O and δ^18^O values is lower in this method due to incomplete combustion of the graphite rod.

**Figure 6:**
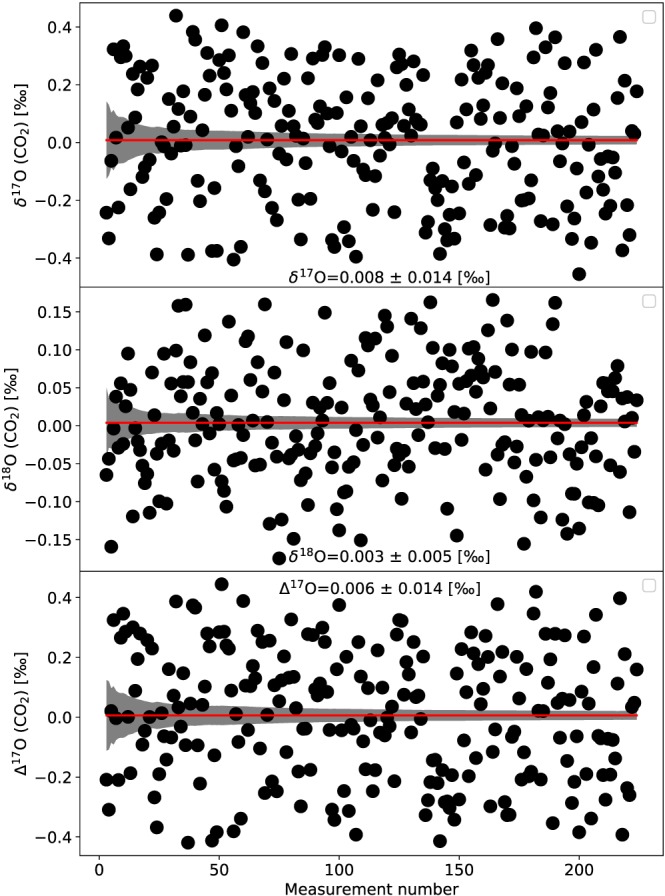
A long‐term zero enrichment experiment (Δ^17^O, δ^17^O and δ^18^O) at the University of Göttingen. After 20 h of measurement time a precision of 14 ppm for δ^17^O and Δ^17^O, and 5 ppm for δ^18^O is achieved [Color figure can be viewed at wileyonlinelibrary.com]

**Figure 7:**
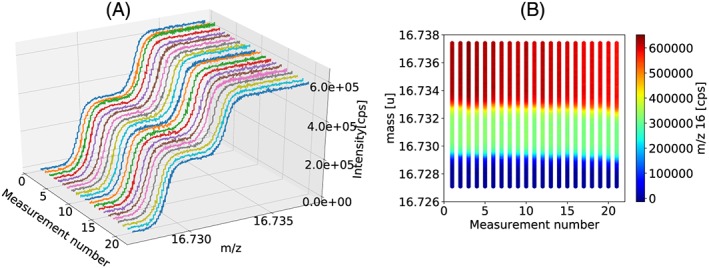
A, Medium‐resolution mass sweep for m/z 17 performed during the isotope measurement to monitor the stability of the mass scale. Each line represents a single mass spectrum that was recorded after each acquisition of 10 cycles of dual‐inlet isotope measurements. The separation between two mass sweeps is roughly 21 min. B, 2‐D projection of A, where the ion count rate is presented in color to show the stability of the plateau used for measurement of the ^17^O^+^ fragment (green section) [Color figure can be viewed at wileyonlinelibrary.com]

Due to the low ion counts very long measurement times are required to achieve a precision of the order of 10 ppm. A long‐term measurement of a zero enrichment cylinder reference gas at the University of Göttingen (Tyczka Industrie‐Gase GmbH, Mannheim, Germany) yielded a precision of 14 ppm for Δ^17^O and δ^17^O values (5 ppm for δ^18^O values) after a measurement time of 20 h (Figure 6). As mentioned above, a requirement is that the mass scale remains very stable over the entire measurement period. At Utrecht University we monitor the stability of the mass scale by recording a medium‐resolution mass spectrum at regular intervals during the measurement. Figures 7A and 7B show an example of a long‐term fragment measurement during which the mass scale was very stable. However, the mass scale is not always as stable, and mass instabilities are one limitation for measurements that require long measurement times. Instabilities in the mass scale are more likely to contribute to the larger errors than counting statistics, factor Γ in Table 3, in some measurements.

- Δ^17^O accuracy

**Figure 8:**
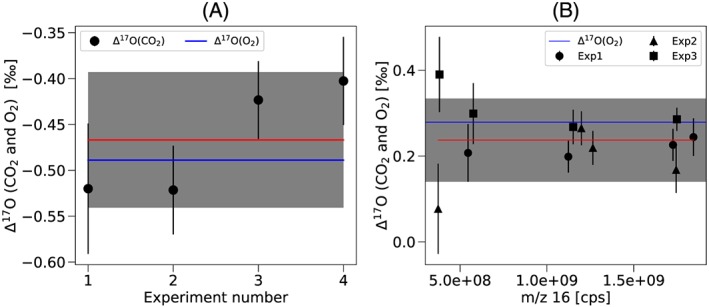
A, Δ^17^O of CO_2_ produced by combustion of a graphite rod (black points and red line showing the mean) and Δ^17^O of the pure O_2_ used for combusting the graphite (blue line), measured at Utrecht University. B, Similar results for CO_2_ that was prepared from isotopically depleted O_2_ at the University of Göttingen, plotted versus the m/z 16 signal intensity. Δ^17^O values obtained from the fragment method are indistinguishable from the Δ^17^O values of the combusted O_2_. The Δ^17^O is calculated using λ = 0.528 for both gases. Individual error bars represent ±1 standard error of the mean (SEM). The shaded area shows the SEM times Student's t‐factor (95% confidence) [Color figure can be viewed at wileyonlinelibrary.com]

The accuracy of Δ^17^O and δ^17^O measurements using the O‐fragment method is evaluated by measuring CO_2_ with known δ^17^O and δ^18^O values, prepared from isotopically known O_2_ (see section 4.5.1) The results presented in Figure 8A and Table 3 show that Δ^17^O of the CO_2_ obtained by measuring the δ^17^O and δ^18^O values from the ^17^O^+^ and ^18^O^+^ fragment ions is indistinguishable within the experimental error from the isotopic composition of the O_2_ used for the preparation of the CO_2_. The assigned Δ^17^O value of the reference O_2_ used for combustion at Utrecht University is −0.489 ± 0.008‰ while the CO_2_ obtained by combustion has Δ^17^O = −0.467 ± 0.074‰ when measured with the fragment method (Figure 8A and Table 3). To enable easy comparison, the Δ^17^O of O_2_ and CO_2_ are both calculated with the same value of λ = 0.528. In addition, the individual δ^17^O and δ^18^O values agree with those of the source O_2_ within the errors. It should be noted that the discrepancy of Δ^17^O results within our measurement series is larger than the errors from the individual measurements, which indicates that sample handling errors have contributed to the rather large spread in the fragment measurements. The isotopically light O_2_ in Göttingen has assigned values of δ^17^O = −26.239 ± 0.002‰ and δ^18^O = −49.614 ± 0.002‰ relative to VSMOW, which yields Δ^17^O = 0.279 ± 0.006‰. The CO_2_ produced by combustion and measured with the O‐fragment method (Figure 8B, Table 3) shows a rather wide range of δ^17^O and δ^18^O values, indicating fractionation (and/or incomplete combustion) in the process of preparing the CO_2_. The effect on Δ^17^O is much smaller.

The good agreement between the δ^17^O, δ^18^O and Δ^17^O values of oxygen and of the CO_2_ produced by combusting graphite shows that determination of the triple isotopic composition of CO_2_ using the O‐fragment method is not only reproducible but also accurate. Furthermore, the agreement in the triple isotopic composition of oxygen between O_2_ and CO_2_ (produced by combustion) suggests that our isotope scales for CO_2_ and O_2_ are very compatible.

As shown in Table S3 (supporting information), Δ^17^O is measured with an average standard error of 39 ppm (standard error of the mean) for four measurements (A_3_, B_2_, B_3_, C_2_) at an intensity for *m/z* 16 of 1.18 × 10^9^ cps. When measurements are made at lower signal intensity than the linear range for source pressure vs signal intensity relation for *m/z* 16 (see above), measurement precision decreases. For instance, the precision drops from 39 to 83 ppm (average SEM for the four measurements shown in Table S3, supporting information) when the intensity on *m/z* 16 decreases from 1.18 × 10^9^ to 4.70 × 10^8^ cps. Measurement at higher signal intensity, outside the linear window, does not show a significant improvement in the precision of the Δ^17^O measurement relative to measurements with lower signal intensity in the linear window (Table S3, supporting information). This might be also due to statistics since we only have four measurements.

- Comparison of the O‐fragment method with the CO_2_‐O_2_ exchange method

**Figure 9:**
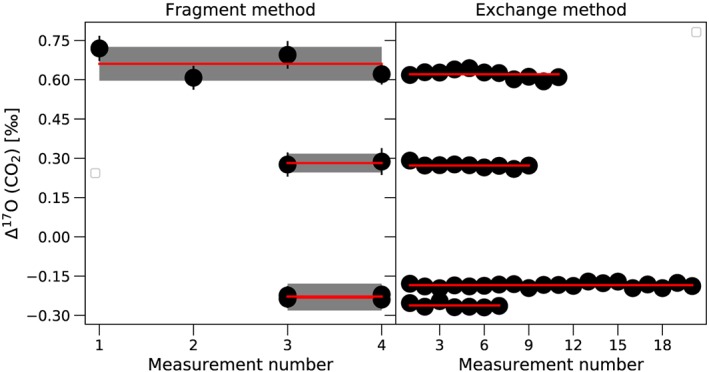
Comparison of Δ^17^O measured with the fragment method and the CO_2_‐O_2_ exchange method for four different CO_2_ gases. The δ^18^O values of the CO_2_ gases range from 4.48‰ to 35.00‰. The horizontal axis shows the number of experiments. Error bars for the fragment measurement represent ±1 standard error of the mean (SE). The red line shows the mean and the shaded area is the standard error of the mean times student t‐factor (95% confidence) [Color figure can be viewed at wileyonlinelibrary.com]

After confirming the accuracy and reproducibly of the O‐fragment method, we measured the δ^17^O, δ^18^O and Δ^17^O values of four CO_2_ gases both with the O‐fragment method and with the oxygen exchange method (see above). Two of the gases are commercial CO_2_ gases (G1 and G2, Table 1) and the other two (G3 and G4) were artificially enriched in ^17^O as described in section 3.5.2. As shown in Figure 9 and Table S4 (supporting information), the results obtained with the two totally independent techniques are indistinguishable within the error bars. The δ^18^O values are in the range of 4.8–35.0‰ vs VSMOW and values of Δ^17^O range from −0.3‰ to +0.7‰ (λ =0.528) which covers and extends the Δ^17^O range expected for tropospheric CO_2_ samples, including international carbonate standards.^32^ The Δ^17^O is determined by the O‐fragment method with a precision of 36–79 ppm (standard error times Student's t‐factor for 95% confidence). The excellent agreement between the two totally independent methods provides an independent validation of the fragment ion technique.

- C‐fragment

The δ^13^C values of the two CO_2_ gases G1 and SCOTT were measured against G2 with the C‐fragment method and with the traditional measurement on the CO_2_ molecule (evaluated with the Brand et al^8^ procedure). As shown in Table 4, the δ^13^C values obtained from the C‐fragment method and molecular measurement are the same within the error (at the ≈ 0.01‰ reproducibility level). A possible challenge for measuring δ^13^C values with the fragment method is the interference from the ^12^CH^+^ adduct due to ion source chemistry (e.g. in the presence of water). The ^12^CH^+^ adduct is only 0.004 u separated from ^13^C^+^ as shown in the mass spectra (Figure 4). However, the figure also shows that this interference can be resolved at medium resolution.

## 4 DISCUSSION

### 4.1 Scale contraction

We observe a higher scale contraction when measuring on the fragment ions than with the measurements on the molecular ions (Table 2). The difference might be because fragment ions are more reactive than the molecular ions. High energy collisions between ions and the source material cause sputtering and implantation, which may be more effective for fragment ions. Therefore, fragment ions may remain effectively longer in the ion source causing the observed higher scale contraction. The difference in scale contraction between fragment measurement and molecular measurement requires further study.

### 4.2 Possible interferences

Oxygen isotope measurements on O fragment ions with low‐resolution mass spectrometers are mainly limited by the interference from water and its OH fragment ions. The background level of water in mass spectrometers is always significant, and it also generally varies when switching between bellows in dual‐inlet measurements. With the 253 Ultra, these interferences can be separated from the O^+^ fragments (Figure 2; Table S5, supporting information), even if the shoulder for interference‐free ^17^O^+^ measurements is narrow. H_2_^16^O^+^ is the main interference for ^18^O^+^ and ^16^OH^+^ for ^17^O^+^. The two rare isotopologues of OH, ^17^OH and ^16^OD, could also interfere with ^18^O, but they are negligible in abundance compared with H_2_^16^O and can be resolved at medium mass resolving power. Table S5 (supporting information) shows a list of other potential interferences with cardinal masses 17 and 18. The molecules made up of lighter atoms than O have masses that are always higher than the cardinal masses 17 and 18, because O is the lightest element where the exact isotope masses are lighter than the cardinal masses. Therefore, these interferences all fall on the high mass side of the O^+^ fragment ion, and they can also be resolved with the 253 Ultra at medium resolution (the mass resolving power required is lower than that for separating OH^+^ and H_2_O^+^). Therefore, only interferences from doubly ionized oxygen formed in the ion source (^16^O^18^O^++^) and other doubly ionized molecules with higher masses (e.g. ^34^S^++^ or ^36^Ar^++^, Table S5, supporting information) can potentially interfere at the low‐mass shoulders where we perform measurements. Formation of doubly ionized ions is usually suppressed by several orders of magnitude compared with the singly charged ions. Nevertheless, they interfere at the low‐mass shoulder of the O atom fragments. The interference of ^16^O^18^O^++^ on ^17^O^+^ depends on the δ^18^O value and source pressure as shown in Figure 3. At a source pressure of 2.5 × 10^−7^ mbar, the size of the correction in our instrument is about 0.5 ppm in the δ^17^O value (and thus Δ^17^O) per 1‰ difference in the δ^18^O value between sample and working reference gas. Thus, when the working reference gas is close in isotopic composition to the samples that are measured, the correction is negligible.

The other challenge to measuring the δ^17^O and δ^18^O values of CO_2_ using the fragment method is the possible interference of O fragment ions from other oxygen‐bearing impurities (OBI) such as H_2_O, O_2_ or N_2_O. The sample and the mass spectrometer background should be very clean to avoid any oxygen contribution from other molecules. The effect of an OBI on the values of δ^17^O, δ^18^O and Δ^17^O measurements of CO_2_ (δ^I^_imp_) can be estimated using Equation 8. The magnitude of the interference depends on the isotopic composition, the fragmentation pattern (efficiency of producing O fragment ions relative to CO_2_), ionization efficiency and the abundance of the impurity relative to the CO_2_ (Equation 8).

(8) $${\delta^{I}}_{\mathrm{imp}}=\psi *\Omega *\rho *\varphi *{\delta^{I}}_{\left(\mathrm{OBI}\;\mathrm{vs}\;{\mathrm{CO}}_{2}\right)}$$

where *I* is 17 or 18, $\rho =\frac{\left[\mathrm{OBI}\right]}{\left[{\mathrm{CO}}_{2}\right]}\;$ is the abundance ratio, *Ω* is the ratio of oxygen atoms in OBI to the oxygen atoms of CO_2_, *ψ* is the ratio in ionization efficiency of OBI to CO_2_ and *φ* is the ratio of O^+^ fragment formation of OBI versus CO_2_. As mentioned above, a water background is always present in mass spectrometers and therefore we estimate the effect of water on the δ^17^O, δ^18^O and Δ^17^O measurements of CO_2_ using Equation 8. For water *Ω* = 0.5 and *φ* = 0.1 because the O^+^ fragment production is only 1% for H_2_O, whereas it is 10% for CO_2_.^67^, ^68^ We assume a similar ionization efficiency between CO_2_ and H_2_O (i.e. *ψ* = 1) for the calculation. Table S6 (supporting information) shows the calculated effect of water impurity on the δ^17^O, δ^18^O and Δ^17^O values of CO_2_ measured with the O‐fragment method for different water levels and isotopic composition of the water. For instance, when the isotopic composition of the water impurity relative to the CO_2_ is δ^17^O = −20‰ and δ^18^O = −40‰, the effect on the δ^17^O and δ^18^O values of CO_2_ will be significant for *ρ* >0.3% and *ρ* >0.1%, respectively. Since the isotopic composition of the water is assumed (roughly) to be mass dependent, the effect on the Δ^17^O will be only significant when *ρ* >1%. When the isotopic composition is strongly mass independent (δ^17^O = δ^18^O = −40‰ relative to CO_2_), the effect on Δ^17^O will be significant for *ρ* >0.3% (Table S6, supporting information).

### 4.3 Future developments and applications

In the present state of development, the O‐fragment method can be used to quantify Δ^17^O of CO_2_ with a precision about of 37 ppm in about 12 h measurement time (67.1 s integration time and 60 s equilibration time). Higher precisions can be achieved by (i) increasing signal intensity; (ii) increasing observation/integration time of the ^17^O^+^ fragment ions (Figure 6); and (iii) achieving measurement precisions at the counting statistics limits. The signal intensity can be increased by increasing source pressure, but the present measurements are already at the upper end of the range where signal intensity increases linearly with source pressure (Figure S1, supporting information). Increasing the ion current will also shorten the filament lifetime. Observation time can be increased by simply extending the integration time, by reducing the time that is used for peak centering, pressure adjust, etc., and by reducing the equilibration time. Reducing the equilibration time introduces additional error due to cross contamination/mixing between sample and reference. Ideally, a LIDI (Long Integration Dual Inlet) technique where the sample‐reference switching is not performed at all would enable longer observation times of the sample.^69^ LIDI measurements were attempted with the 253 Ultra but not continued because of instability issues. An increase in stability may also enable measurements at the counting statistics limit, which would improve precision by a factor of 1.5.

Compared with traditional δ^13^C measurements that require a ^17^O‐correction, the C‐fragment is not subject to the following uncertainties related to the ^17^O‐correction:

1. The use of different ^17^R, ^13^R and λ values in different algorithms introduces discrepancies that are larger than the precision of current isotope ratio mass spectrometry techniques^42^
2. Most of the correction algorithms used do not include the impact of Δ^17^O of CO_2_
3. The accepted values for ^17^R and ^13^R may require revision to meet the current measurement precision^44^
4. There is no single λ value that can be assigned to CO_2_ since different processes that contribute to the formation or removal of CO_2_ follow different three‐isotope slopes.

The fragment technique is simple and unlike other techniques does not require any additional chemical conversion or exchange steps to measure the δ^17^O value of CO_2_. Therefore, it can be used to independently assess discrepancies in δ^17^O values measured by different laboratories, such as the difference in δ^17^O of IAEA (International Atomic Energy Agency, Vienna, Austria) carbonate standard (NBS‐18) measured by Passey et al^32^ and Barkan et al.^7^ However, the signal intensities for rare isotopes of fragment ions are relatively small, especially when they have to be separated from near‐by mass interferences and require higher mass resolution, which reduces ion transmission in the 253 Ultra. Therefore, long measurement times are required to reach a precision of the order of 0.01‰. When this precision is reached, the fragment technique can also be useful to evaluate discrepancies introduced in δ^13^C measurements due to the use of different algorithms for ^17^O‐correction.

Isotope measurements of atomic ion fragments may have many applications for other molecules. A straightforward extension of the application presented here is the mass‐interference‐free measurement of ^17^O^+^ and ^18^O^+^ in other oxygen‐containing compounds, for example, CO or N_2_O. Current isotope techniques of these gases rely in many cases on an assumed relation of mass‐dependent fractionation between δ^17^O and δ^18^O values and (e.g. in the case of the CO) chemical conversion into CO_2_.^70^, ^71^, ^72^ Direct isotope ratio measurements on the O^+^ fragment can overcome these limitations and provide quantification of Δ^17^O. Similar to the case of CO_2_ presented here, the ^13^C^+^ content of CH_4_ and CO can be measured directly on the C^+^ fragment of these gases, without chemical conversion steps that are known to cause artifacts in traditional isotope techniques.^70^, ^71^, ^72^, ^73^ Furthermore, isotope measurements on atomic fragment ions may be combined with measurements of larger fragments of hydrocarbons to determine the position‐specific carbon isotope composition of hydrocarbons.^55^

The position‐specific ^15^N^+^ content of N_2_O is presently determined by measurement of the parent N_2_O molecule and the NO fragment, which allow the average δ^15^N value and the ^15^N content at the central nitrogen position to be quantified, and the δ^15^N value of the terminal N atom is derived by mass balance, which induces large errors.^51^, ^52^ In principle, the ^15^N^+^ content of the terminal N atom could be derived from the N^+^ fragment, which originates primarily from the terminal N atom in N_2_O. Similar to the case of O atoms shown here, this requires a very good vacuum system to avoid contamination from the main atmospheric gas N_2_.

In addition to these environmental applications, the analysis of atomic fragment ions of different compounds may be a useful tool to study fractionation processes in the ion source of an isotope ratio mass spectrometer. As discussed earlier, the scale contractions for isotopic measurements are different for the fragment ions and molecular ions of CO_2_. Examining these effects further may help to understand the chemistry and surface effects in the ion source of isotope ratio mass spectrometers by studying different fragments. In addition, analysis of fragment ions facilitates measuring the isotopic composition of two different chemical compounds versus each other (e.g. δ^13^C value in CH_4_ versus in CO_2_). This can on the one hand provide information on ion source effects associated with fragmentation, but on the other it may also help to directly compare isotope scales between different compounds.

## Supporting information


**Figure S1**
Relationship between source pressure and signal intensity at m/z 16 (Faraday collector L3, equipped with a 1x10^10^Ω resistor). The linear range (shaded area) ends at a source pressure of 4.5x10^−7^ mbar (corresponding to a signal intensity of approx. 1.3x10^9^ cps).
**Figure S2** Schematic diagram of the O_2_‐CO_2_ exchange experimental setup. The quartz reactor has an outer diameter of 21 mm. PID stands for proportional‐integral‐derivative temperature controller.
Figure S3 Schematic diagram of the setup used for conversion of O_2_ to CO_2_ by combusting a graphite rod. The reactor is made out of quartz with 21 mm outer diameter.
Figure S4 Schematic diagram of the setup used for preparing CO_2_ with a positive Δ^17^O by photolysing a mixture of O_2_ and CO with UV light. BR: Borosilicate reactor (2 L), PRL: pen ray Hg vapor lamp, CT: CO_2_ trap (liquid nitrogen temperature), SV: sample vial, WT: water trap (operated at dry ice temperature), PS: pressure sensor, HVP: high vacuum pump, FVP: fore vacuum pump.
**Figure S5** δ^13^C and δ^18^O values of SCOTT measured against G2. A and B) Effect of equilibration time with VISC window closed. C and D) Effect of equilibration time with VISC window fully opened; E and F) Effect of filament emission current on the relative difference between the two gases in δ^13^C and δ^18^O values. G and H) Effect of amount of gas in the ion source quantified by the signal intensity in cps for m/z 44. The emission current experiments are performed at 60‐seconds equilibration time and the sensitivity to the amount of gas was determined with 30‐seconds equilibration time. The VISC window was kept closed.
**Figure S6** Effect of the equilibration time on the δ^13^C (A) and δ^18^O (B) differences between measurement with open VISC window (VISCo) and closed VISC window (VISCc).
**Figure S7** Drop in signal intensity for at m/z 44 (corresponding to the source pressure) when the dual inlet valve was closed for different initial source pressures. A) decrease of the main ion signal of CO_2_ as a function of time. B) fraction of CO_2_ remaining as a function of time for the main ion signal of CO_2_. The emission current and accelerating voltage were 1.95 mA and 9.9 kV, respectively.
**Figure S8** Comparison of δ^17^O differences between three different CO_2_ gases with the fragment technique using the 253 Ultra at Utrecht University The red arrows indicate that the respective measured δ values are combined for comparison with the directly measured third δ value.
**Figure S9** Comparison of δ^18^O differences between three different CO_2_ gases using either the molecular CO_2_ ion technique (left) or the atom fragment technique (right) with the 253 Ultra at Utrecht University The red arrows indicate that the respective measured δ values are combined for comparison with the directly measured third δ value.
**Table S1** Effect of equilibration time on the absolute isotopic difference of two gases measured on the 253 Ultra with VISC window open and closed. The errors given are standard errors of the mean.
**Table S2** Effect of emission control current and source pressure on the absolute isotopic difference of two gases measured on the 253 Ultra (G1 and SCOTT). The errors given are standard errors of the mean. The integration time of individual measurements is 61.7 seconds The effects of source pressure (intensity of m/z 44) are determined at an equilibration time of 30 seconds and an emission current of 1.8 mA.
**Table S3** Isotopic composition of CO_2_ produced by combustion with isotopically light O_2_ at the University of Göttingen. Each CO_2_ sample is analyzed four times at different intensity to investigate the effect of signal intensity on the precision of Δ^17^O measurement based on the relationship between source pressure and signal intensity at m/z 16.
**Table S4**: Comparison of the results obtained with the O_2_‐CO_2_ exchange method and O‐fragment technique. δ^17^O, δ^18^O and Δ^17^O values are in per mill (‰) with respect to VSMOW. The error of the mean is the standard error multiplied by the student t‐factor for the 95% two‐sided confidence interval while for the individual measurements it is the standard error. Γ is the ratio between measured precision and the precision calculated according to counting statistics, n is number of cycles, i stand for initial (before exchange) and f stands for final (after exchange). For δ^18^O values the measurement error is similar to the error calculated based on counting statistics.
**Table S5** List of potentially interfering ions with masses close to ^17^O^+^ and ^18^O^+^, and the required resolution to avoid the interference. The interfering ions are ordered based on the resolving power requirement.
**Table S6** Simulated effect of a H_2_O impurity (contamination level quantified by γ = [H_2_O]/[CO_2_] as source of oxygen during Δ^17^O measurement using the O‐fragment method for an intensity for m/z 16 of 4.05x10^9^ cps. The signal of O atom fragments relative to molecular ions is 10% for CO_2_ and 1% for H_2_O^5,6^. For these conceptual calculations we assumed the same ionization efficiency for H_2_O and CO_2_.
