# Supplementary material for: Determination of the triple oxygen and carbon isotopic composition of CO2 from atomic ion fragments formed in the ion source of the 253 Ultra high‐resolution isotope ratio mass spectrometer
Source: Rapid Commun Mass Spectrom. 2019 Aug 5;33(17):1363–80. doi: 10.1002/rcm.8478 (PMC6771542; doi:10.1002/rcm.8478)
Supplement: Supplementary file 1 — Figure S1 Relationship between source pressure and signal intensity at m/z 16 (Faraday collector L3, equipped with a 1x10^10^Ω resistor). The linear range (shaded area) ends at a source pressure of 4.5x10^−7^ mbar (corresponding to a signal intensity of approx. 1.3x10^9^ cps). Figure S2 Schematic diagram of the O2‐CO2 exchange experimental setup. The quartz reactor has an outer diameter of 21 mm. PID stands for proportional‐integral‐derivative temperature controller. Figure S3 Schematic diagram of the setup used for conversion of O2 to CO2 by combusting a graphite rod. The reactor is made out of quartz with 21 mm outer diameter. Figure S4 Schematic diagram of the setup used for preparing CO2 with a positive Δ17O by photolysing a mixture of O2 and CO with UV light. BR: Borosilicate reactor (2 L), PRL: pen ray Hg vapor lamp, CT: CO2 trap (liquid nitrogen temperature), SV: sample vial, WT: water trap (operated at dry ice temperature), PS: pressure sensor, HVP: high vacuum pump, FVP: fore vacuum pump. Figure S5 δ13C and δ18O values of SCOTT measured against G2. A and B) Effect of equilibration time with VISC window closed. C and D) Effect of equilibration time with VISC window fully opened; E and F) Effect of filament emission current on the relative difference between the two gases in δ13C and δ18O values. G and H) Effect of amount of gas in the ion source quantified by the signal intensity in cps for m/z 44. The emission current experiments are performed at 60‐seconds equilibration time and the sensitivity to the amount of gas was determined with 30‐seconds equilibration time. The VISC window was kept closed. Figure S6 Effect of the equilibration time on the δ13C (A) and δ18O (B) differences between measurement with open VISC window (VISCo) and closed VISC window (VISCc). Figure S7 Drop in signal intensity for at m/z 44 (corresponding to the source pressure) when the dual inlet valve was closed for different initial source pressures. A) decrease of the main ion signal of CO [file RCM-33-1363-s001.docx]

Determination of the triple oxygen and carbon isotopic composition of CO_2_ from atomic ion fragments formed in the ion source of the 253 Ultra High-Resolution Isotope Ratio Mass Spectrometer

Getachew A. Adnew^1*^, Magdalena E.G. Hofmann^1**^, Dipayan Paul^1,2^, Amzad Laskar^1^, Jakub Surma^3^, Nina Albrecht^3***^, Andreas Pack^3^, Johannes Schwieters^4^, Gerbrand Koren^5^, Wouter Peters^2,5^ and Thomas Röckmann^1^

^1^ Institute for Marine and Atmospheric research Utrecht (IMAU), Utrecht University, The Netherlands

^2^ Centre for Isotope Research, University of Groningen, The Netherlands

^3^ Geoscience Center Göttingen, Georg-August-University Göttingen, Germany

^4^ Thermo Fisher Scientific, Bremen, Germany

^5^ Department of Meteorology and Air Quality, Wageningen University, The Netherlands

** Now at Picarro Inc, Santa Clara, CA, USA

*** Now at Thermo Fisher Scientific, Bremen, Germany

*Corresponding author: g.a.adnew@uu.nl

**Effect of emission control current, source pressure, equilibration time and source conductance on the scale contraction**

In order to characterize the 253 Ultra instrument, we performed an extensive investigation of factors that are known to affect the scale contraction in isotope ratio mass spectrometers (Verkouteren et al^1,2^) , in particular the ion source pressure (quantified by signal intensity), the filament current and the equilibration time after switching the changeover valve. The experimental results are provided in **Tables S1 - S2** and **Figures S5 - S7**, and discussed below. The δ^18^O and δ^13^C values obtained from the molecular ion measurements are calculated using the algorithm presented in Brand et al^3^.

When the VISC window is closed, the source pressure of 2.5x10^-7^ mbar corresponds to an ion count rate of 3x10^11^ cps for *m/z* 44, which reduces to 8.0 x10^9^ cps when the VISC window is fully open (the signal reduces to about 3%). As shown in **Figure S5 A** and **5 B**, with the VISC window open, the measurement with equilibration time of 10 seconds results in a scale contraction of -36 ppm for δ^13^C values and -33 ppm for δ^18^O values compared with measurements at 90-seconds equilibration time. The scale contractions are smaller at 20-seconds equilibration time, and for equilibration times ≥30 seconds we do not find a significant scale contraction compared with measurements with 90-seconds equilibration time for both δ^13^C and δ^18^O values. When the VISC window is closed, the effect of equilibration time is much higher: at an equilibration time of 10 seconds, the scale is contracted by -79 ppm and -71 ppm for δ^13^C and δ^18^O values, respectively, relative to a measurement at 90-seconds equilibration time. Again, the scale contraction reduces with longer equilibration times, but only at an equilibration time of ≥50 seconds; the scale contraction does not change significantly any more with longer equilibration times. In general, the cross-contamination coefficient is higher for δ^18^O measurements than for δ^13^C measurements as shown in **Figure S5 A** and **5 B (VISC closed)** and **5 C** and **5 D** (VISC fully open) for different equilibration time, and **5 E** and **5 F** for different emission current.

The relative difference between δ^13^C measurements with VISC open and VISC closed decreases from 0.110 ‰ to 0.066 ‰ when the equilibration time increases from 10 seconds to 90 seconds (**Figure S6 A).** The corresponding relative difference for δ^18^O measurements changes from 0.125‰ to 0.088 ‰ (**Figure S6 B).**

Measurements at a filament emission current of 1.95 mA cause a scale contraction of -21 ppm in δ^13^C values and -39 ppm in δ^18^O values relative to measurements at an emission current of 1 mA (**Figures S5 C** and **5 D**). At 1.5 mA emission current, the scale contraction relative to the measurement at 1 mA emission current is -8 ppm for δ^13^C values while the difference between measurements at 1.5 mA and 1 mA emission current is insignificant for δ^18^O values.

The source pressure (quantified using the signal intensity under otherwise constant conditions) can also influence the scale contraction. A decrease in the intensity of *m/z* 44 from 2.5x10^11^ cps to 9x10^10^ cps causes a scale contraction of -39 ppm and -35 ppm for δ^13^C and δ^18^O respectively (**Figure S5 E** and **5 F)***.*

One factor that can be responsible for a scale contraction is that a fraction of the previous gas remains in the ion source when the changeover valve is switched. This effect is often called cross contamination. To obtain an indication of gas exchange rates when switching between different gases, we monitored the pressure drop when the changeover valve was closed. The percentage of the gas remaining in the ion source with time depends on the initial source pressure or signal intensity (at *m/z* 44) in the ion source as shown in **Figure S7**. As expected, the source is evacuated much more quickly when the VISC window is open. The loss rate of CO_2_ from the ion source can be approximated mathematically by a double exponential function (**Figure S7**). The first exponential term is interpreted as a time scale for the pumping of gas out of the ion source, while the second exponential may reflect timescales of surface interactions in the ion source. At lower source pressure (lower count *m/z* 44) the absolute amount of gas remaining in the source with time is lower than at higher ion source pressure (higher count *m/z* 44). However, the relative amount of the gas in the source with respect to the initial source pressure is higher when the ion source pressure is lower which explains partly why we have larger scale contraction /memory effect when the ion source pressure, or signal intensity, is lower (**Figures S5 E** and **5 F**). At higher ion source pressure, the relative effect of the remaining gas is smaller than at lower source pressure.

As shown in **Figures S5 C** and **5 D,** when the emission control current increases the absolute relative difference in δ^18^O and δ^13^C values between the two gases decreases (scale contraction). Verkouteren et al^1,2^ showed that a higher emission current results in higher sputtering and implantation of ions in the source slit material. This causes adsorption and desorption of molecules from the surface and to the surface resulting in considerable memory effect (cross contamination**^4^** or mixing of reference and sample)^1,2^. These scale contraction effects depend on the material of the source slit and are lowest for a tantalum slit. Although the source slit in the 253 Ultra is made from tantalum we still observe a scale contraction at equilibration times of 15 second unlike the observation reported by Verkouteren et al^1,2^. This might partly be due to the difference in measurement conditions (source pressure, accelerating voltage, emission current, etc.), differences in the source housing and differences in tuning parameters between the MAT 253 and 253 Ultra instruments.

**Figure S1** Relationship between source pressure and signal intensity at *m/z* 16 (Faraday collector L3, equipped with a 1x10^10^Ω resistor). The linear range (shaded area) ends at a source pressure of 4.5x10^-7^ mbar (corresponding to a signal intensity of approx. 1.3x10^9^ cps).

**Figure S2** Schematic diagram of the O_2_-CO_2_ exchange experimental setup. The quartz reactor has an outer diameter of 21 mm. PID stands for proportional-integral-derivative temperature controller.

Figure S3 Schematic diagram of the setup used for conversion of O_2_ to CO_2_ by combusting a graphite rod. The reactor is made out of quartz with 21mm outer diameter.

Figure S4 Schematic diagram of the setup used for preparing CO_2_ with a positive Δ^17^O by photolysing a mixture of O_2_ and CO with UV light. BR: Borosilicate reactor (2 L), PRL: pen ray Hg vapor lamp, CT: CO_2_ trap (liquid nitrogen temperature), SV: sample vial, WT: water trap (operated at dry ice temperature), PS: pressure sensor, HVP: high vacuum pump, FVP: fore vacuum pump.

**Figure S5** δ^13^C and δ^18^O values of SCOTT measured against G2. A and B) Effect of equilibration time with VISC window closed. C and D) Effect of equilibration time with VISC window fully opened; E and F) Effect of filament emission current on the relative difference between the two gases in δ^13^C and δ^18^O values. G and H) Effect of amount of gas in the ion source quantified by the signal intensity in cps for *m/z* 44. The emission current experiments are performed at 60-seconds equilibration time and the sensitivity to the amount of gas was determined with 30-seconds equilibration time. The VISC window was kept closed.

**Figure S6** Effect of the equilibration time on the δ^13^C (A) and δ^18^O (B) differences between measurement with open VISC window(VISCo) and closed VISC window (VISCc).

**Figure S7** Drop in signal intensity for at *m/z* 44 (corresponding to the source pressure) when the dual inlet valve was closed for different initial source pressures. A) decrease of the main ion signal of CO_2_ as a function of time. B) fraction of CO_2_ remaining as a function of time for the main ion signal of CO_2_. The emission current and accelerating voltage were 1.95 mA and 9.9 kV, respectively.

**Figure S8** Comparison of δ^17^O differences between three different CO_2_ gases with the fragment technique using the 253 Ultra at Utrecht University The red arrows indicate that the respective measured δ values are combined for comparison with the directly measured third δ value.

**Figure S9** Comparison of δ^18^O differences between three different CO_2_ gases using either the molecular CO_2_ ion technique (left) or the atom fragment technique (right) with the 253 Ultra at Utrecht University The red arrows indicate that the respective measured δ values are combined for comparison with the directly measured third δ value.

**Figure S8** Comparison of δ^13^C differences between three different CO_2_ gases using either the molecular CO_2_ ion technique (left) or the atom fragment technique (right) with the 253 Ultra mass spectrometer at Utrecht University. The red arrows indicate that the respective measured δ values are combined for comparison with the directly measured third δ value.

**Table S1** Effect of equilibration time on the absolute isotopic difference of two gases measured on the 253 Ultra with VISC window open and closed. The errors given are standard errors of the mean.

| Equilibration time (s) | VISC window closed | | VISC window open | |
| --- | --- | --- | --- | --- |
|  | δ^13^C values (‰) | δ^18^O values (‰) | δ^13^C values (‰) | δ^18^O values (‰) |
| 10 | -36.7184±0.001 | -20.1901±0.001 | -36.8246±0.001 | -20.3126±0.002 |
| 20 | -36.7587±0.001 | -20.2263±0.002 | -36.8492±0.001 | -20.3305±0.002 |
| 30 | -36.7685±0.001 | -20.2324±0.002 | -36.8675±0.001 | -20.3447±0.002 |
| 40 | -36.7758±0.001 | -20.2397±0.002 | -36.8543±0.001 | -20.3399±0.002 |
| 50 | -36.7834±0.001 | -20.2510±0.001 | -36.8597±0.001 | -20.3411±0.0022 |
| 60 | -36.7858±0.001 | -20.2541±0.002 | -36.8594±0.001 | -20.3406±0.0012 |
| 90 | -36.7949±0.001 | -20.2592±0.002 | -36.8590±0.0012 | -20.3450±0.002 |

**Table S2** Effect of emission control current and source pressure on the absolute isotopic difference of two gases measured on the 253 Ultra (G1 and SCOTT). The errors given are standard errors of the mean. The integration time of individual measurements is 61.7 seconds The effects of source pressure (intensity of *m/z* 44) are determined at an equilibration time of 30 seconds and an emission current of 1.8 mA.

| Effect of emission current on CO_2_ isotopic composition | | | |
| --- | --- | --- | --- |
| Equilibration time (s) | Emission control current (mA) | δ^13^C values (‰) | δ^18^O values (‰) |
| 60 | 1.00 | -36.8679±0.0013 | -20.3443±0.0023 |
| 60 | 1.00 | -36.8694±0.0013 | -20.3351±0.0029 |
| 60 | 1.00 | -36.8668±0.0014 | -20.3372±0.0029 |
| 60 | 1.50 | -36.8589±0.0010 | -20.3399±0.0019 |
| 60 | 1.50 | -36.8608±0.0011 | -20.3340±0.0015 |
| 60 | 1.50 | -36.8596±0.0009 | -20.3373±0.0016 |
| 60 | 1.50 | -36.8635±0.0009 | -20.3413±0.0018 |
| 60 | 1.95 | -36.8371±0.0011 | -20.2943±0.0018 |
| 60 | 1.95 | -36.8389±0.0008 | -20.2975±0.0016 |
| 60 | 1.95 | -36.8443±0.0010 | -20.3072±0.0016 |
| Effect of intensity of *m/z* 44 on CO_2_ isotopic composition | | | |
| Equilibration time (s) | *m/z* 44(cps) | δ^13^C values (‰) | δ^18^O values (‰) |
| 30 | 2.5E+11 | -36.712±0.001 | -20.184±0.001 |
| 30 | 1.5E+11 | -36.689±0.001 | -20.160±0.001 |
| 30 | 9E+10 | -36.675±0.001 | -20.150±0.002 |

**Table S3** Isotopic composition of CO_2_ produced by combustion with isotopically light O_2_ at the University of Göttingen. Each CO_2_ sample is analyzed four times at different intensity to investigate the effect of signal intensity on the precision of Δ^17^O measurement based on the relationship between source pressure and signal intensity at *m/z* 16.

| Experiment | δ^17^O values (‰) | δ^18^O values (‰) | Δ^17^O (‰) | *m/z* 16 [cps] |
| --- | --- | --- | --- | --- |
| Measurement in the linear range of signal (*m/z* 16) vs source pressure | | | | |
| A3 | -26.940±0.037 | -50.764±0.038 | 0.199±0.037 | 1.12E+09 |
| B2 | -26.465±0.038 | -50.005±0.022 | 0.265±0.039 | 1.20E+09 |
| B3 | -26.278±0.052 | -49.579±0.054 | 0.219±0.039 | 1.26E+09 |
| C2 | -26.453±0.040 | -49.989±0.014 | 0.268±0.040 | 1.15E+09 |
| Average standard error | | | ±0.039 | 1.18E+09 |
| Measurement outside the linear range, at higher signal intensity than the linear range | | | | |
| A1 | -27.103±0.069 | -51.148±0.013 | 0.244±0.044 | 1.84E+09 |
| A2 | -27.094±0.03 | -51.099±0.014 | 0.226±0.037 | 1.73E+09 |
| B1 | -26.676±0.053 | -50.222±0.016 | 0.168±0.054 | 1.74E+09 |
| B1 | -26.484±0.027 | -50.078±0.014 | 0.286±0.027 | 1.75E+09 |
| Average standard error | | | ±0.041 | 1.77E+09 |
| Measurement outside the linear range, at lower signal intensity than the linear range | | | | |
| A4 | -26.977±0.066 | -50.848±0.041 | 0.207±0.067 | 5.46E+08 |
| B4 | -26.981±0.104 | -50.623±0.072 | 0.077±0.105 | 3.74E+08 |
| C3 | -26.469±0.067 | -50.074±0.030 | 0.299±0.071 | 5.76E+08 |
| C4 | -26.0766±0.083 | -49.514±0.031 | 0.390±0.088 | 3.82E+08 |
| Average standard error | | | ±0.083 | 4.70E+08 |

**Table S4**: Comparison of the results obtained with the O_2_-CO_2_ exchange method and O-fragment technique. δ^17^O, δ^18^O and Δ^17^O values are in per mill (‰) with respect to VSMOW. The error of the mean is the standard error multiplied by the student t-factor for the 95% two-sided confidence interval while for the individual measurements it is the standard error. Γ is the ratio between measured precision and the precision calculated according to counting statistics, *n* is number of cycles, *i* stand for initial (before exchange) and *f* stands for final (after exchange). For δ^18^O values the measurement error is similar to the error calculated based on counting statistics.

| Sample code | n | Γ | Fragment technique | | | CO_2_-O_2_ exchange method | | | | | | |
| --- | --- | --- | --- | --- | --- | --- | --- | --- | --- | --- | --- | --- |
|  |  |  |  |  |  | O_2_ | | | | CO_2_ | | |
|  |  |  | δ^17^O | δ^18^O | Δ^17^O | δ^17^O_i_ | δ^18^O_i_ | δ^17^O_f_ | δ^18^O_f_ | δ^17^O_i_ | δ^18^O_i_ | Δ^17^O**_i_** |
| G4 | 119 | 1.6 | 19.987±0.049 | 36.778±0.013 | 0.719±0.048 | 9.254 | 18.542 | 14.409 | 27.456 | 19.891 | 36.793 | 0.618 |
|  | 110 | 1.5 | 19.882±0.046 | 36.796±0.012 | 0.608±0.046 | 9.254 | 18.542 | 14.111 | 26.933 | 19.902 | 36.793 | 0.628 |
|  | 110 | 1.7 | 19.974±0.053 | 36.803±0.013 | 0.695±0.053 | 9.254 | 18.542 | 14.156 | 27.011 | 19.900 | 36.793 | 0.627 |
|  | 128 | 1.4 | 19.895±0.040 | 36.795±0.012 | 0.621±0.040 | 9.254 | 18.542 | 14.187 | 27.054 | 19.913 | 36.793 | 0.639 |
|  | **19.935±0.069** | | | **36.793±0.012** | **0.661±0.064** | 9.254 | 18.542 | 14.047 | 26.808 | 19.918 | 26.808 | 0.644 |
|  |  |  |  |  |  | 9.254 | 18.542 | 14.139 | 26.981 | 19.901 | 26.981 | 0.627 |
|  |  |  |  |  |  | 9.254 | 18.542 | 14.035 | 26.803 | 19.899 | 36.793 | 0.625 |
|  |  |  |  |  |  | 9.254 | 18.542 | 14.036 | 26.825 | 19.874 | 36.793 | 0.601 |
|  |  |  |  |  |  | 9.254 | 18.542 | 14.113 | 26.950 | 19.884 | 36.793 | 0.611 |
|  |  |  |  |  |  | 9.254 | 18.542 | 14.033 | **26.826** | 19.866 | 36.793 | 0.594 |
|  |  |  |  |  |  | 9.254 | 18.542 | 14.039 | 26.823 | 19.882 | 36.793 | 0.610 |
| **Mean**  **±SE*t** |  |  |  |  |  |  | | | | **19.894±0.009** |  | **0.620±0.008** |
|  | | | | | | | | | | | | |
| G3 | 96 | 1.4 | 19.161±0.046 | 36.058±0.013 | 0.277±0.046 | 9.254 | 18.542 | 13.751 | 26.562 | 19.181 | 36.067 | 0.291 |
|  | 95 | 1.6 | 19.182±0.052 | 36.076±0.014 | 0.288±0.051 | 9.254 | 18.542 | 13.788 | 26.646 | 19.161 | 36.067 | 0.272 |
|  | **19.183±0.067** | | |  | **0.282±0.036** | 9.254 | 18.542 | 13.719 | 26.521 | 19.163 | 36.067 | 0.273 |
|  |  |  |  |  |  | 9.254 | 18.542 | 13.729 | 26.537 | 19.166 | 36.067 | 0.276 |
|  |  |  |  |  |  | 9.254 | 18.542 | 13.867 | 26.785 | 19.163 | 36.067 | 0.273 |
|  |  |  |  |  |  | 9.254 | 18.542 | 13.683 | 26.464 | 19.154 | 36.067 | 0.265 |
|  |  |  |  |  |  | 9.254 | 18.542 | 13.594 | 26.300 | 19.160 | 36.067 | 0.271 |
|  |  |  |  |  |  | 9.254 | 18.542 | 13.659 | 26.426 | 19.148 | 36.067 | 0.259 |
|  |  |  |  |  |  | 9.254 | 18.542 | 13.677 | 26.447 | 19.161 | 36.067 | 0.272 |
| **Mean**  **±SE*t** |  |  |  |  |  |  | | | | **19.162±0.005** |  | **0.273±0.005** |
|  | | | | | | | | | | | | |
| G1 | 145 | 1.2 | 2.354±0.035 | 4.889±0.013 | -0.224±0.036 | 9.254 | 18.542 | 5.560 | 11.344 | 2.399 | 4.843 | -0.179 |
|  | 143 | 1.1 | 2.339±0.034 | 4.891±0.013 | -0.240±0.035 | 9.254 | 18.542 | 5.560 | 11.354 | 2.389 | 4.843 | -0.190 |
|  | **2.347±0.046** | | | **4.890±0.007** | **-0.232±0.049** | 9.254 | 18.542 | 5.545 | 11.331 | 2.383 | 4.843 | -0.196 |
|  |  |  |  |  |  | 9.254 | 18.542 | 5.502 | 11.237 | 2.393 | 4.843 | -0.185 |
|  |  |  |  |  |  | 9.254 | 18.542 | 5.592 | 11.416 | 2.389 | 4.843 | -0.189 |
|  |  |  |  |  |  | 9.254 | 18.542 | 5.511 | 11.256 | 2.392 | 4.843 | -0.187 |
|  |  |  |  |  |  | 9.254 | 18.542 | 5.522 | 11.273 | 2.396 | 4.843 | -0.182 |
|  |  |  |  |  |  | 9.254 | 18.542 | 5.473 | 11.176 | 2.398 | 4.843 | -0.181 |
|  |  |  |  |  |  | 9.254 | 18.542 | 5.485 | 11.214 | 2.383 | 4.843 | -0.196 |
|  |  |  |  |  |  | 9.254 | 18.542 | 5.525 | 11.280 | 2.395 | 4.843 | -0.184 |
|  |  |  |  |  |  | 9.254 | 18.542 | 5.567 | 11.362 | 2.395 | 4.843 | -0.184 |
|  |  |  |  |  |  | 9.254 | 18.542 | 5.685 | 11.595 | 2.391 | 4.843 | -0.187 |
|  |  |  |  |  |  | 9.254 | 18.542 | 5.680 | 11.571 | 2.408 | 4.843 | -0.171 |
|  |  |  |  |  |  | 9.254 | 18.542 | 5.576 | 11.373 | 2.402 | 4.843 | -0.177 |
|  |  |  |  |  |  | 9.254 | 18.542 | 5.578 | 11.372 | 2.408 | 4.843 | -0.171 |
|  |  |  |  |  |  | -20.85 | -38.20 | -9.749 | -17.492 | 2.358 | 4.843 | -0.196 |
|  |  |  |  |  |  | -20.85 | -38.20 | -9.611 | -17.247 | 2.372 | 4.843 | -0.182 |
|  |  |  |  |  |  | -20.85 | -38.20 | -9.451 | -16.936 | 2.358 | 4.843 | -0.196 |
|  |  |  |  |  |  | -20.85 | -38.20 | -9.463 | -16.977 | 2.378 | 4.843 | -0.176 |
|  |  |  |  |  |  | -20.85 | -38.20 | -9.619 | -17.257 | 2.366 | 4.843 | -0.188 |
| **Mean±SE*t** |  |  |  |  |  |  | | | | **2.388±0.006** |  | **-0.185±0.003** |
|  | | | | | | | | | | | | |
| G2 | 145 | 1.2 | 18.054±0.035 | 34.931±0.013 | -0.236±0.035 | 9.254 | 18.542 | 13.271 | 26.155 | 18.071 | 34.998 | -0.253 |
|  |  |  |  |  |  | 9.254 | 18.542 | 13.253 | 26.134 | 18.057 | 34.998 | -0.267 |
|  |  |  |  |  |  | 9.254 | 18.542 | 13.281 | 26.167 | 18.079 | 34.998 | -0.246 |
|  | 143 | 1.1 | 18.068±0.04 | 34.929±0.013 | -0.220±0.034 | 9.254 | 18.542 | 13.349 | 26.318 | 18.055 | 34.998 | -0.269 |
|  |  | | | | | 9.254 | 18.542 | 13.234 | 26.098 | 18.057 | 34.998 | -0.267 |
|  |  |  |  |  |  | 9.254 | 18.542 | 13.247 | 26.125 | 18.055 | 34.998 | -0.269 |
|  |  |  |  |  |  | 9.254 | 18.542 | 13.233 | 26.093 | 18.060 | 34.998 | -0.264 |
| **Mean±SE*t** | **18.061±0.047** | | | **34.930±0.007** | **-0.228±0.049** |  | | | | **18.062±0.007** |  | **-0.262±0.007** |

**Table S5** List of potentially interfering ions with masses close to ^17^O^+^ and ^18^O^+^, and the required resolution to avoid the interference. The interfering ions are ordered based on the resolving power requirement.

| Interfering ion | Exact mass [u] | Mass difference [mu] | Required resolution [m/Δm] |
| --- | --- | --- | --- |
| **^17^O^+^** | **16.9991** | | |
| ^16^O^18^O ^++^ | 16.9970 | 2.1 | 8118 |
| ^16^OH^+^ | 17.0027 | 3.6 | 4712 |
| ^34^S^++^ | 16.9839 | 15.2 | 1118 |
| ^15^NH_2_^+^ | 17.0158 | 16.6 | 1023 |
| ^14^NHD^+^ | 17.0250 | 25.9 | 658 |
| ^13^CH_2_D^+^ | 17.0331 | 34.0 | 501 |
| ^13^CH_4_^+^ | 17.0347 | 35.5 | 479 |
| ^13^CD_2_H^+^ | 17.0360 | 36.9 | 461 |
| ^12^CH_3_D^+^ | 17.0376 | 38.4 | 443 |
| ^12^CH_5_^+^ | 17.0391 | 40.0 | 426 |
| **^18^O^+^** | **17.9992** | | |
| ^17^OH^+^ | 18.0070 | 7.8 | 2309 |
| ^16^OD^+^ | 18.0090 | 9.9 | 1827 |
| ^16^OH_2_^+^ | 18.0106 | 11.4 | 1579 |
| ^36^Ar^++^ | 17.9838 | 15.4 | 1169 |
| ^15^NH_3_^+^ | 18.0236 | 24.4 | 737 |
| ^14^NH_4_^+^ | 18.0344 | 35.2 | 512 |
| ^14^CH_4_^+^ | 18.0345 | 35.4 | 509 |
| ^13^CDH_3_^+^ | 18.0409 | 41.8 | 431 |
| ^13^CH_5_^+^ | 18.0425 | 43.3 | 416 |
| ^12^CD_2_H_2_^+^ | 18.0439 | 44.7 | 403 |
| ^12^CDH_4_^+^ | 18.0454 | 46.2 | 390 |

**Table S6** Simulated effect of a H_2_O impurity (contamination level quantified by γ = [H_2_O]/[CO_2_] as source of oxygen during Δ^17^O measurement using the O-fragment method for an intensity for *m/z* 16 of 4.05x10^9^ cps. The signal of O atom fragments relative to molecular ions is 10% for CO_2_ and 1 % for H_2_O^5,6^. For these conceptual calculations we assumed the same ionization efficiency for H_2_O and CO_2_.

| $\rho$ | O fragment from water | | | O fragment of the mixture | | | Isotopic composition | | |
| --- | --- | --- | --- | --- | --- | --- | --- | --- | --- |
|  | ^16^O^+^ | ^17^O^+^ | ^18^O^+^ | ^16^O^+^ | ^17^O^+^ | ^18^O^+^ | δ^17^O | δ^18^O | Δ^17^O |
| δ^17^O and δ^18^O values of water vs CO_2_ are -20 and -40 ‰, respectively [mass dependent] | | | | | | | | | |
| 1.00E-04 | 2.00E+05 | 7.47E+01 | 3.95E+02 | 4.05E+09 | 1.54E+06 | 8.33E+06 | -0.001 | -0.002 | 0.000 |
| 3.00E-04 | 6.00E+05 | 2.24E+02 | 1.18E+03 | 4.05E+09 | 1.54E+06 | 8.33E+06 | -0.003 | -0.006 | 0.000 |
| 1.00E-03 | 2.00E+06 | 7.47E+02 | 3.95E+03 | 4.06E+09 | 1.54E+06 | 8.33E+06 | -0.010 | **-0.020** | 0.001 |
| 3.00E-03 | 6.00E+06 | 2.24E+03 | 1.18E+04 | 4.06E+09 | 1.55E+06 | 8.34E+06 | **-0.030** | **-0.059** | 0.002 |
| 1.00E-02 | 2.00E+07 | 7.47E+03 | 3.95E+04 | 4.07E+09 | 1.55E+06 | 8.37E+06 | **-0.098** | **-0.196** | 0.006 |
| 3.00E-02 | 6.00E+07 | 2.19E+04 | 1.18E+05 | 4.11E+09 | 1.57E+06 | 8.45E+06 | **-0.584** | **-0.584** | **-0.276** |
| δ^17^O and δ^18^O values of water vs CO_2_ are -40 and -40 ‰, respectively [mass independent] | | | | | | | | | |
| 1.00E-04 | 2.00E+05 | 7.32E+01 | 3.95E+02 | 4.05E+09 | 1.54E+06 | 8.33E+06 | -0.002 | -0.002 | -0.001 |
| 3.00E-04 | 6.00E+05 | 2.19E+02 | 1.18E+03 | 4.05E+09 | 1.54E+06 | 8.33E+06 | -0.006 | -0.006 | -0.003 |
| 1.00E-03 | 2.00E+06 | 7.32E+02 | 3.95E+03 | 4.06E+09 | 1.54E+06 | 8.33E+06 | -0.020 | **-0.020** | -0.009 |
| 3.00E-03 | 6.00E+06 | 2.19E+03 | 1.18E+04 | 4.06E+09 | 1.55E+06 | 8.34E+06 | **-0.059** | **-0.059** | **-0.028** |
| 1.00E-02 | 2.00E+07 | 7.32E+03 | 3.95E+04 | 4.07E+09 | 1.55E+06 | 8.37E+06 | **-0.196** | **-0.196** | **-0.093** |

References

1. Verkouteren RM, Allison CE, Studley SA, Leckrone KJ. Isotopic metrology of carbon dioxide. I. Interlaboratory comparison and empirical modeling of inlet equilibration time, inlet pressure, and ion source conductance. *Rapid Commun Mass Spectrom.* 2003;17(8):771-776.

2. Verkouteren RM, Assonov S, Klinedinst DB, Brand WA. Isotopic metrology of carbon dioxide. II. Effects of ion source materials, conductance, emission, and accelerating voltage on dual-inlet cross contamination. *Rapid Commun Mass Spectrom.* 2003;17(8):777-782.

3. Brand WA, Assonov SS, Coplen TB. Correction for the ^17^O interference in δ(^13^C) measurements when analyzing CO_2_ with stable isotope mass spectrometry (IUPAC Technical Report). *Pure Appl Chem.* 2010;82(8):1719-1733.

4. Assonov SS, Brenninkmeijer CA. A new method to determine the ^17^O isotopic abundance in CO_2_ using oxygen isotope exchange with a solid oxide. *Rapid Commun Mass Spectrom.* 2001;15(24):2426-2437.

5. NIST. Carbon dioxide-the NIST webbook. *NIST chemistry webbook,SRD 69* 2018; <https://webbook.nist.gov/cgi/cbook.cgi?ID=C124389&Mask=200>. Accessed December 17, 2018.

6. NIST. Mass spectrum (electron ionization)-the NIST webbook. *NIST chemistry webbook,SRD 69* 2018; <https://webbook.nist.gov/cgi/cbook.cgi?ID=C7732185&Mask=200>. Accessed December 17, 2018.
